# Reindeer control over subarctic treeline alters soil fungal communities with potential consequences for soil carbon storage

**DOI:** 10.1111/gcb.15722

**Published:** 2021-06-14

**Authors:** Henni Ylänne, Rieke L. Madsen, Carles Castaño, Daniel B. Metcalfe, Karina E. Clemmensen

**Affiliations:** ^1^ Centre for Environmental and Climate Science Lund University Lund Sweden; ^2^ Department of Biology Lund University Lund Sweden; ^3^ Uppsala BioCenter Department of Forest Mycology and Plant Pathology Swedish University of Agricultural Sciences Uppsala Sweden; ^4^ Department of Physical Geography and Ecosystem Science Lund University Lund Sweden; ^5^ Department of Ecology and Environmental Sciences Umeå University Umeå Sweden

**Keywords:** Arctic shrubification, *Betula pubescens* ssp. *czerepanovii*, fungal community, grazing, ITS2, *Rangifer tarandus*, subarctic tundra, tree‐line

## Abstract

The climate‐driven encroachment of shrubs into the Arctic is accompanied by shifts in soil fungal communities that could contribute to a net release of carbon from tundra soils. At the same time, arctic grazers are known to prevent the establishment of deciduous shrubs and, under certain conditions, promote the dominance of evergreen shrubs. As these different vegetation types associate with contrasting fungal communities, the belowground consequences of climate change could vary among grazing regimes. Yet, at present, the impact of grazing on soil fungal communities and their links to soil carbon have remained speculative. Here we tested how soil fungal community composition, diversity and function depend on tree vicinity and long‐term reindeer grazing regime and assessed how the fungal communities relate to organic soil carbon stocks in an alpine treeline ecotone in Northern Scandinavia. We determined soil carbon stocks and characterized soil fungal communities directly underneath and >3 m away from mountain birches (*Betula pubescens* ssp. *czerepanovii*) in two adjacent 55‐year‐old grazing regimes with or without summer grazing by reindeer (*Rangifer tarandus*). We show that the area exposed to year‐round grazing dominated by evergreen dwarf shrubs had higher soil C:N ratio, higher fungal abundance and lower fungal diversity compared with the area with only winter grazing and higher abundance of mountain birch. Although soil carbon stocks did not differ between the grazing regimes, stocks were positively associated with root‐associated ascomycetes, typical to the year‐round grazing regime, and negatively associated with free‐living saprotrophs, typical to the winter grazing regime. These findings suggest that when grazers promote dominance of evergreen dwarf shrubs, they induce shifts in soil fungal communities that increase soil carbon sequestration in the long term. Thus, to predict climate‐driven changes in soil carbon, grazer‐induced shifts in vegetation and soil fungal communities need to be accounted for.

## Graphical abstract

We assessed soil fungal community composition and its links to soil carbon storage in two contrasting long‐term reindeer grazing regimes in the subarctic treeline ecotone. Root‐associated basidiomycetes and free‐living moulds and yeasts were characteristic to the regime with only wintertime grazing, whereas few taxa of root‐associated ascomycota dominated fungal communities on the open heaths in the year‐round grazing regime. These patterns in fungal functional guild abundance were linked to organic soil carbon storage: root‐associated ascomycetes were more abundant in plots with high soil carbon storage, whereas saprotrophic guilds were typical to plots with low soil carbon storage.

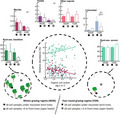

## 1 INTRODUCTION

Arctic and subarctic regions are rapidly warming, which is leading to northward and altitudinal range expansion of shrubs and trees (Myers‐Smith et al., 2019; Tape et al., 2012; Terskaia et al., 2020). Although this ‘shrubification’ likely contributes to higher primary production (Zhang et al., 2014), it could also accelerate fungal‐driven soil organic matter (OM) decomposition and result in a large net loss of ecosystem carbon and exacerbate climate change (Hartley et al., 2012; Parker et al., 2015). At the same time, virtually all of the Arctic experiences various degrees of vertebrate and invertebrate herbivory (Barrio et al., 2016; Rheubottom et al., 2019), which is known to hamper tree and shrub establishment and growth (Dahlgren et al., 2009; Kumpula et al., 2011; Sundqvist et al., 2019), induce tree mortality (Jepsen et al., 2013) and keep tree‐lines lower than their altitudinal limits (Cairns & Moen, 2004; Oksanen et al., 1995; Van Bogaert et al., 2011). Thus, to truly predict the realized vegetation ranges and ecosystem processes in warmer temperatures, the past, current and future herbivore densities need to be accounted for (Schmitz et al., 2018).

Reindeer (*Rangifer tarandus* L.), also known as the caribou in North America, is the most wide‐spread vertebrate herbivore in the Arctic (Bernes et al., 2015) with wild or semi‐domesticated reindeer herds roaming over the vast majority of Arctic tundra (Conservation of Arctic Flora and Fauna (CAFF), 2001). These animals adapt to the pronounced seasonality of the Arctic by altering their metabolic efficiency between seasons and by migrating large distances to optimize forage intake according to availability (Bernes et al., 2015). During winters, reindeer lichens (*Cladonia*) are a central nutrition source for most reindeer herds, whereas during summers, reindeer browse on the leaves of shrubs and deciduous trees and graze on graminoids (grasses, sedges and rushes) and herbs. Here, we refer to the combined effects of both foraging behaviours as grazing. Depending on the season and reindeer abundance, reindeer may alter tundra vegetation and, in certain conditions, even shift the ecosystem to an alternative state with altered process rates and limited capacity to recover to the earlier state (Egelkraut et al., 2018; van der Wal, 2006; Ylänne et al., 2020). Observations of reindeer‐induced state shifts have led to suggestions that reindeer management (Bråthen et al., 2017) and even reintroductions of large grazers (Olofsson & Post, 2018) could be used to reduce some of the unwanted effects of a warmer climate, particularly the shrubification. However, the consequences of such interventions on soil carbon storage remain largely uncertain (Olofsson & Post, 2018) and are likely dependent on the characteristics of the ecosystem, such as the dominant plant traits (sensu Wardle et al., 2004). For example, ecosystems dominated by fast‐growing plants with high nitrogen content are likely to benefit from the grazing‐induced high return of labile fecal material to the soil, leading to herbivory promoting faster soil nutrient turnover and higher productivity rates (Wardle et al., 2004). In contrast, ecosystems dominated by slow‐growing plants could respond to herbivory by increasing the amount of defence compounds, which would reduce litter quality and ultimately lead to reduced soil nutrient availability and productivity in grazed systems (Wardle et al., 2004). Field surveys from the tundra show evidence of reindeer‐induced shifts in both directions, with either positive (Egelkraut et al., 2018; Olofsson et al., 2004; Stark & Väisänen, 2014) or negative (Sundqvist et al., 2019; Vowles et al., 2017; Ylänne et al., 2015) consequences on plant palatability and nitrogen content. Which one of these alternative scenarios takes place is likely to determine grazing impacts on soil microbial communities and the ecosystem processes they regulate, such as soil carbon sequestration (Wardle et al., 2004).

Grazers' control over soil carbon sequestration could be particularly important in situations where grazing favours the dominance of evergreen shrubs over deciduous trees and shrubs. This could specifically be relevant in Fennoscandia where the deciduous genus *Betula* has been suggested to be key for ecosystem carbon storage (Hartley et al., 2012; Parker et al., 2018). Although the high‐stature *Betula* species store large amounts of carbon in their biomass, total ecosystem carbon storage is lower in both mountain birch (*Betula pubescens* ssp. *czerepanovii*) forests and dwarf birch (*B. nana*) shrublands compared with evergreen shrub‐dominated tundra heaths due to lower soil carbon stocks in *Betula*‐dominated ecosystems (Hartley et al., 2012; Parker et al., 2018). The differences in soil carbon have been suggested to be linked to the mycorrhizal fungi associated with the dominant vegetation (Parker et al., 2015; Vowles & Björk, 2019; Clemmensen et al., 2021). Although ectomycorrhizal fungi (EcM) associated with birch may generally grow and turn over biomass fast, many of the ericoid mycorrhizal fungi (ErM) associated with evergreen shrubs have melanised cell walls that are relatively resistant to decomposition (Clemmensen et al., 2015; Fernandez & Kennedy, 2018; Read & Perez‐Moreno, 2003). In addition, certain EcM fungi are known to efficiently access and mine nitrogen from OM by using oxidative enzymes (Bödeker et al., 2014; Kyaschenko et al., 2017) and by traits adapted to long‐distance nutrient transport, such as cords (Clemmensen et al., 2015). This contributes to EcM‐dominated ecosystems generally maintaining higher rates of OM decomposition—and lower rates of humus accumulation—compared with ecosystems dominated by ErM fungi (Clemmensen et al., 2015, 2021). The difference between EcM‐dominated mountain birch forests and ErM‐dominated tundra heaths could be particularly strong if the difference in dominant mycorrhizal association is accompanied by a shift in free‐living saprotrophic activity (Vowles & Björk, 2019). Due to the higher litter quality of deciduous versus evergreen leaves (Cornwell et al., 2008) and the soil community adapted to this (Parker et al., 2018), the activity of saprotrophic fungi is generally higher in mountain birch forests compared with tundra heaths.

Given what is known about the role of herbivores in climate‐driven vegetation changes and their potential to promote an evergreen shrub–dominated plant community, herbivores' potential to increase tundra soil carbon stocks via promoting the dominance of ErM has been proposed (Vowles & Björk, 2019), yet evidence for this mechanism is lacking. Here, we test this idea across two adjacent grazing regimes, which have experienced either only winter grazing or year‐round grazing by reindeer due to long‐standing differences in Finnish and Norwegian reindeer husbandry. The winter‐only grazing regime is characterized by a low‐stature mountain birch woodland, whereas summer browsing in the year‐round grazing regime (YGR) has hindered tree establishment (Biuw et al., 2014; Kumpula et al., 2011) turning this area into a savannah‐like open tundra heath with individual mountain birches scattered very sparsely in the landscape (Oksanen et al., 1995). Here, we firstly investigate the impacts of grazing regime and mountain birch vicinity on the abundance, diversity and community composition of the soil fungal community and secondly explore how the soil fungal communities relate to vegetation and, ultimately to soil carbon stocks. Specifically, we test the hypotheses, that (H1) year‐round grazing by reindeer and greater distance to mountain birch trees increase the relative abundance of ErM, while decreasing EcM and saprotrophic fungi, and that (H2) ErM‐dominated communities are associated with higher organic soil carbon stocks than EcM‐dominated communities, particularly if (H3) the EcM fungi belong to the cord‐forming exploration type and/or if (H4) the mycorrhizal shift from EcM to ErM is accompanied with lower abundance of free‐living saprotrophs.

## 2 MATERIALS AND METHODS

### 2.1 Study site, experimental design and soil sampling

The study was carried out at the tree‐line ecotone in the area of Jávrrešduoddarat at the Norwegian‐Finnish border (68°45ʹN, 23°43ʹE; altitude 430–480 m a.s.l.), where the mean annual temperature is −1.4℃ and the mean annual precipitation is 530 mm (2010–2019; Data from the Norwegian Meteorological Institute; www.senorge.no). The landscape consists of mountain birch forests, shrublands and tundra heaths on uplands and fens in depressions (Kitti et al., 2009). In the late 1950's, a border fence was built to separate the reindeer herds of the two countries (Forbes & Kumpula, 2009). Due to the migratory herding tradition in Norway, where reindeer move between coastal summer grazing grounds and inland winter grazing grounds, the Norwegian side of the Jávrrešduoddarat area is grazed only during snow‐covered winter months. In contrast, the Finnish side of the Jávrrešduoddarat area is grazed all year round with the grazing pressure in summer being approximately 10–12 reindeer per square kilometre (Kitti et al., 2009). As reindeer exert less control over vegetation during winter months, when soil and vegetation are mostly protected by snow and ice (Kumpula et al., 2011), the year‐round grazed side is more exposed to reindeer browsing, trampling and fertilization by excreta than the winter grazed side (Forbes & Kumpula, 2009). This long‐term grazing difference is reflected in higher lichen cover in the winter grazing regime (WGR) compared with the YGR (Cohen et al., 2013; Forbes & Kumpula, 2009) and in the abundance of mountain birch (*B. pubescens* ssp. *czerepanovii* (Orlova) Hämet‐Ahti) at the treeline ecotone (Oksanen et al., 1995). Although ~70 years of summer browsing by reindeer in the YGR has hampered regeneration of mountain birch stands (Biuw et al., 2014) and resulted in scattered trees occurring sparsely in a savannah‐like open landscape, the WGR is characterized by high abundance of mountain birch (Oksanen et al., 1995).

Here, we compared the open woodland in the YGR to the denser mountain birch woodlands in the WGR by sampling three 350 × 200 m sized blocks, separated by ~1 km, along the border fence. All blocks had one side in the YGR and one side in the WGR, with no difference in topography, altitude or slope between the two sides of the fence. The average densities of mountain birch were 213 ± 115 and 3 ± 2 trees ha^−1^ in the WGR and YGR, respectively, with slightly taller (3.3 ± 0.5 m), one‐ or two‐stemmed trees in the YGR and shorter (2.6 ± 0.8 m), multi‐stemmed trees in the WGR (see Figure S1). In both grazing regimes, the field layer was dominated by evergreen and deciduous dwarf shrubs, such as *Empetrum nigrum* L. ssp. *hermaphroditum* (Hagerup) Böcher, *B. nana* L., *Calluna vulgaris* (L.) Hull, *Phyllodoce caerulea* (L.) Bab. and *Vaccinium* L. species. In each block, six coordinates were marked on either side of the fence, 20 m from the fence with 50 m between neighbouring points (Figure S2a). At each coordinate, we collected soil samples below the canopy of the nearest mountain birch and the nearest point that was at least three meters away from the nearest mountain birch (Figure S2b), where the low‐stature shrubs dominated the vegetation. This study design amounted to a total of 72 samples (3 blocks × 2 grazing regimes × 6 coordinates × 2 distances to mountain birch trees). As this study is based on a singular border fence that separates the grazing regimes of the neighbouring countries, a risk of pseudoreplication affecting the results exists. Yet, we have minimized potential dependencies caused by this design by keeping a higher distance between consecutive plots on each side of the fence than between the plot pairs across the fence (Olofsson et al., 2004).

Soil samples were collected on the 28th and 29th of September 2019 by taking three to four soil cores (Ø 5 cm) from the upper 5 cm of the soil. The sample included the litter and humus layers in each plot, and both live vegetation and mineral soil was carefully removed, and the depth of each sample was measured. While sampling, hummocks were avoided. As hummocks typically form around tree‐stems, the soil cores close to mountain birches were collected below the outer border of the canopy. Within 48 h of collection, soil samples were weighed and divided into two equally sized halves and stored at −18℃. One half of the soil sample was used to determine soil water (SWC, drying at 105℃ for >18 h) and OM content (loss on ignition at 405℃, 5 h). The other half was freeze‐dried (LYO GT2; LYOVACTM; GEA Group) and mixed manually during which large roots and stones were removed. A subset (2 ml) of the mixed soil was homogenized with a ball mill (MM200; Retsch) before DNA extraction and chemical analyses. Soil carbon and nitrogen contents were analysed with vario Max CN (Elementar) and used to calculate organic soil C:N ratio and C and N stocks (SOC and SON, kg m^−2^).

### 2.2 DNA extraction and quantitative PCR (qPCR)

Total DNA was extracted from 100 mg of dried soil, using the NucleoSpin® Soil kit (Macherey‐Nagel) and the DNA concentration was estimated spectrophotometrically (NanoDrop 2000c; Thermo Scientific) and diluted 1:20. Polymerase chain reaction (PCR) inhibition was tested by spiking each sample with a known amount of the pGEM‐16S plasmid (Promega) and amplifying a region on the plasmid using the primers M13F and M13R on a CFX Connect Real‐Time System (Bio‐Rad). No inhibition was detected in the samples. Copy numbers of the fungal ITS2 region were then quantified in duplicates of all samples using the fungal primers gITS7 (Ihrmark et al., 2012), ITS4 (White et al., 1990) and ITS4arch (Sterkenburg et al., 2018) on the same Real‐Time System. The reaction mix (20 μl) consisted of 0.5 μM gITS7, 0.3 μM ITS4, 0.15 μM ITS4arch, 0.1% bovine serum albumin, 1 × iQ‐SYBR Green (Bio‐Rad) and 2 μl of diluted DNA template. The PCR cycling conditions were an initial denaturation at 95℃ for 5 min, followed by 40 cycles of denaturation at 95℃ for 15 s, annealing at 56℃ for 30 s, synthesis at 72℃ for 40 s and at 78℃ for 5 s. Standard curves were based on serial dilutions of linearized plasmids with cloned ITS2 fragments from *Pilidium concavum*. Total ITS2 copy numbers were corrected for the proportion of non‐fungal (mainly plant) reads based on sequencing of the same region (see below).

### 2.3 Soil fungal community

The fungal ITS2 region was PCR‐amplified in duplicates from each sample using the same primers as above. All primers were extended by a linker base (T), a unique 8‐base identification tag (differing in at least three positions) and a terminal base (C; Clemmensen et al., 2016). Amplification was performed in a 2720 Thermal Cycler (Life Technologies) following a protocol with reduced numbers of PCR cycles to minimize length biases (Castaño et al., 2020). The 50 μl reaction mix consisted of 0.5, 0.3, 0.15 μM of the gITS7, ITS4 and ITS4arch primers, respectively, 200 μM of each nucleotide, 2.75 mM MgCl_2_ and 0.025 U μl^−1^ of Dream Taq polymerase (Thermo Fisher Scientific). Cycling conditions were an initial denaturation at 95℃ for 5 min, followed by 22–31 cycles of 95℃ for 30 s, 56℃ for 30 s and 72℃ for 30 s, and a final extension step at 72℃ for 7 min. Negative controls (sterile water) were included. Amplicons were cleaned with Agencourt AMPure XP kit (Beckman Coulter, Brea, CA, USA), quantified with Qubit dsDNA HS Assay Kit (Life Technologies), pooled in equal amounts and the pool cleaned using the EZNA Cycle Pure kit (Omega Bio‐Tek). Length distribution of the mix was assessed using a BioAnalyzer 2100 (Agilent Technologies) with a 7500 DNA chip. Samples were sequenced at SciLifeLab NGI with PacBio Sequel I (Pacific Biosciences) using one single‐molecule real‐time cell, after addition of sequencing adapters by ligation.

### 2.4 Bioinformatic analyses

The raw sequence data, containing 372,213 reads, were quality‐filtered and clustered using the SCATA pipeline (https://scata.mykopat.slu.se). ITS2 sequences of <200 bases in length, with an average quality score of less than 20 or containing individual bases with a quality score of less than 7 were removed. Sequences were screened for primers and identification tags and reverse complemented if necessary, and sequences with less than 90% primer or 100% identification tag match were removed. In total, 223,814 sequences passed the quality control. After the collapse of homopolymers to 3 bp, sequences were clustered into species‐level operational taxonomical units (OTUs) using single‐linkage clustering (*U*_SEARCH_; Edgar, 2010) with a threshold distance of 1.5% (Lindahl et al., 2013), mismatch penalty of 1, gap open penalty of 0, gap extension penalty of 1 and end gap penalty of 0 in the pair‐wise comparisons. Genotypes occurring only once in the whole data set were also removed along with sequences with non‐matching sample tags. In total, we obtained 160,683 high‐quality, non‐unique reads which clustered into 992 OTUs.

For taxonomic identification, reference sequences from the UNITE database (Abarenkov et al., 2010) and relevant reference data sets publicly available in SCATA were included in the clustering in SCATA. These provided identifications based on the same criteria as used for the clustering, without affecting it. In addition, the most common genotype of each OTU was subjected to taxonomic assignment in a PROTAX analysis with 50% probability in the PlutoF platform (Abarenkov et al., 2018). Based on taxonomic affiliation and match to references associated with particular substrates (Clemmensen et al., 2013), OTUs were separated into the following functional guilds: root‐associated ascomycetes, root‐associated basidiomycetes, moulds, yeasts, other saprotrophs (including litter‐associated fungi) and lichenized fungi. The root‐associated fungi were further classified into EcM (including all fungal OTUs forming ectomycorrhizal symbiosis) and ErM (excluding the OTUs that also form EcM). EcM fungi were further subdivided according to exploration type (Agerer, 2006) to cord‐formers and simple mycelia as defined by Clemmensen et al. (2015).

After removal of non‐fungal OTUs, 106,872 reads remained. To reduce the risk of random errors induced by multi‐primer artefacts (Tedersoo et al., 2018), all observations with four reads or less were omitted; subsequently, four reads were subtracted from each of the remaining OTUs to restore proportionality of the rarest species in relation to the rest of the community. This resulted in a final data set with a total of 82,845 reads and 364 OTUs, and an average of 1167 (222–8106) reads per sample, used in subsequent statistical analyses. In total, 61% of the OTUs could be assigned to a functional guild, representing 86% of all reads.

### 2.5 Estimated plant abundance

A vegetation survey was conducted on the study area, but it did not evaluate tree abundance and ground layer cover in the same plots as used for soil sample collection. Therefore, we used plant reads captured by the ITS2 sequencing as a proxy of plant root abundance in the sampled soils (see a comparison of vegetation survey and DNA‐based data in the Method S1 description). The plant reads were converted to reads per g DW soil by multiplying their relative abundance by the total ITS2 copy numbers (qPCR) in each sample, and classified into the following groups: *Betula* spp., *Vaccinium* spp., other ericaceous dwarf shrubs (*E. hermaphroditum*, *P. caerulea* and *C. vulgaris*) and bryophytes (*Polytrichum* and *Dicranum* spp.). These four groups contributed 89.0 ± 6.1% of plant cover in the blocks (see Method S1).

### 2.6 Statistical analysis

All statistical analyses were conducted using R version 3.6.3 (R Core Team, 2020) using the package *ggplot2* (v 3.3.0; Wickham, 2016) for data visualization. To assess alpha‐diversity across samples, the package *vegan* (v2.5‐5; Oksanen et al., 2019) was used to calculate the following diversity indices: Species richness, Shannon–Wiener index (*H*), Simpson concentration (*D*) and Pielous' evenness index, using the read data rarefied to minimum sequence read number (*n* = 222). Shannon–Wiener and Simpson indices were further converted to indicate the effective number of species (i.e. Shannon–Wiener index is expressed as exponential function, exp(*H*) and Simpson index as the inverse, 1/*D*; Jost, 2006), and the effective Shannon–Wiener index was used in the calculation of Pielous' evenness index. We then tested for grazing and tree vicinity impacts on the diversity indices, plant and fungal abundance (qPCR) and soil properties using a linear mixed model (package *nlme* v3.1.144; Pinheiro et al., 2014) with block as a random factor. In the case of an interaction, pairwise differences were tested as least square means (package *lsmeans* v2.30.0; Lenth, 2016) using Tukey's adjustment of *p*‐values.

To test the impacts of grazing regime, tree vicinity and their interaction on the relative abundance of taxonomic groups (phyla, subphyla, classes, orders and species), functional guilds and orders and species within functional guilds, multivariate generalized linear models (package *mvabund* v4.1.3; Wang et al., 2012) were built with block set as strata. Non‐rarefied data was used for calculating relative abundances, these were rounded up to closest 0.01% and only sub‐groups with a relative abundance of more than 0.5% were included in the tests. Models were fitted with a negative binomial distribution using 999 bootstrap iterations and reported with log‐likelihood ratio statistics. To test the impacts of grazing regime, tree vicinity and their interaction on finer functional groupings, the previous models were repeated by first replacing the root‐associated guilds with the two mycorrhizal types, and subsequently by replacing EcM fungi with the two exploration types. In all functional guild models, the model was first run including the unknown function or type, and subsequently without the unknown groups (Table S1). As the initial test on taxonomy and functions showed strongest responses on order and species levels with no added impact of mycorrhizal and exploration type (Table S1), only orders and species were tested within the five functional guilds. To reveal grazing and tree impacts on the sub‐groups, the multivariate generalized linear models were followed by univariate tests with unadjusted *p*‐values (summarized for phyla, order and species in Table S2, for functional guilds in Table S3, and for orders and species within the functional guilds in Table S4).

Impacts of grazing regime and tree vicinity on fungal community composition were further visualised using non‐metric multidimensional scaling (NMDS) of Hellinger‐transformed input data using the packages *phyloseq* (v1.30.0; McMurdie & Holmes, 2013) and *vegan* (v2.5–5; Oksanen et al., 2019). Grazing and tree vicinity effects on the community were tested with permutational multivariate ANOVA (*adonis*) using Bray–Curtis distance and 999 permutations. The function *betadisper* was used to assess multivariate homogeneity of group dispersions and no differences induced by grazing regime, tree vicinity or their interaction were observed. We also tested how selected environmental variables, fungal guilds, and orders within saprotrophic and root‐associated guilds (relative abundance > 0.1) explained the community composition identified by NMDS (*envfit*; full list of results in Table S5).

To better understand the connections between the plant and fungal communities, we assessed how the captured abundance of *Betula* sp., *Vaccinium* sp., other ericaceous species and bryophytes correlated with fungal guilds, mycorrhizal types and EcM exploration types (Table S6). For the analysis, all the saprotrophic guilds, that is, moulds, yeasts and other saprotrophs, were merged into one common group of saprotrophic fungi. All correlations included Hellinger‐transformed relative abundances of the fungal groups (out of total fungal reads) and square root‐transformed abundances of plant reads per DW soil.

To test whether fungal abundance and community composition explained soil organic carbon (SOC) stocks, linear models of SOC with fungal abundance (log‐transformed ITS2 copy numbers) and fungal functional groups (Hellinger‐transformed) as explanatory factors were built. The saprotrophic guilds, that is, moulds, yeasts and other saprotrophs, were merged for this analysis. To shed light on the taxonomical changes behind the functional guild shifts, we further inspected correlation among SOC and fungal order and species, limited to groups with relative abundance > 0.1. With all response variables, we first tested how the abundances, and the fungal functional and taxonomic groups explained SOC (Table S7) and then explored whether the explanatory power of the model was increased when effects of grazing and tree vicinity and their interaction were taken into account (i.e. test of within‐treatment relations). Regression assumptions of the models were assessed visually and the model with best explanatory value was selected using the second order Akaike's information criterion (AICc; package AICcmodavg v2.2.2; Mazerolle, 2019). Best explaining models are reported in Table S8.

## 3 RESULTS

### 3.1 Vegetation and soil characteristics in the study area

Based on the captured plant reads in the soil samples, the YGR was characterised by higher abundance of evergreen ericaceous dwarf shrubs, that is, *E. hermaphroditum*, *P. caerulea* and *C. vulgaris*, and bryophytes compared with the WGR (Table 1). The soil organic layer in both grazing regimes was on average 3 cm thick, and soil bulk density, water content and OM content did not differ between the grazing regimes (Table 1; Figure S3). Yet, soil C:N ratio was higher in the YGR (average ± SD 27.8 ± 2.8) compared with the WGR (25.3 ± 3.3; *F*_1,65_ = 11.63, *p* = 0.001). Regardless of grazing regime, soil samples collected in the vicinity of mountain birch were characterised by higher abundance of *Betula* and *Vaccinium* reads and higher soil OM content (Figure S3; Table 1).

**TABLE 1:** Linear mixed‐model results on the impact of summer grazing, tree vicinity and their interaction on the organic soil layer, soil C and N stocks, vegetation abundance, fungal abundance and diversity. Significant effects are bolded and arrows indicate increase (↑) or decrease (↓) towards the year‐round grazing regime or closer vicinity to mountain birch. In case of a significant interaction between grazing regime and tree vicinity; a least square means post hoc test was performed, and the pair‐wise differences are found in the footnotes

	Grazing		Tree		Grazing × Tree	
	*F*_df_	*p*	*F*_df_	*p*	*F*_df_	*p*
Organic soil depth (cm)	1.12_1,65_	0.294	1.68_1,65_	0.199	0.09_1,65_	0.767
Bulk density (g cm^−3^)	2.40_1,65_	0.126	1.00_1,65_	0.320	0.51_1,65_	0.478
Soil organic matter content (OM, %)	0.40_1,65_	0.126	**5.67**_1,65_	**0.020**↑	1.71_1,65_	0.196
Soil water content (SWC, %)	0.02_1,65_	0.889	3.10_1,65_	0.083	0.44_1,65_	0.512
Soil C:N ratio	**11.63**_1,65_	**0.001↑**	1.15_1,65_	0.288	0.33_1,65_	0.565
Soil C stock (kg C m^−2^)	2.55_1,65_	0.115	3.15_1,65_	0.081	0.21_1,65_	0.646
Soil N stock (kg N m^−2^)	0.41_1,65_	0.523	2.48_1,65_	0.120	0.32_1,65_	0.576
*Betula* sp. (ITS2 copy nr g^−1^ DW)	2.89_1,65_	0.094	**7.85_1,65_**	**0.007↑**	0.31_1,65_	0.580
*Vaccinium* sp. (ITS2 copy nr g^−1^ DW)	0.25_1,65_	0.618	**15.35_1,65_**	**<0.001↑**	0.78_1,65_	0.381
Other ericaceous (ITS2 copy nr g^−1^ DW)	**6.85_1,65_**	**0.011↑**	0.15_1,65_	0.703	1.15_1,65_	0.288
Bryophytes (ITS2 copy nr g^−1^ DW)	**16.53_1,65_**	**<0.001↑**	1.26_1,65_	0.267	1.71_1,65_	0.196
Fungal abundance (ITS2 copy nr g^−1^ DW)	**4.96_1,65_**	**0.030↑**	0.30_1,65_	0.584	1.89_1,65_	0.174
Fungal abundance (ITS2 copy nr m^−2^)	**4.29_1,65_**	**0.042↑**	0.45_1,65_	0.505	2.60_1,67_	0.112
Species richness	**6.81_1,65_**	**0.011↓**	0.00_1,65_	0.970	1.83_1,65_	0.181
Species evenness, *J*′	**4.41_1,65_**	**0.040↓**	0.39_1,65_	0.532	3.55_1,67_	0.064
Effective Shannon diversity, exp(*H*)	**5.41_1,65_**	**0.023↓**	0.15_1,65_	0.704	3.52_1,67_	0.065
Inverse Simpson index, 1/*D*	**4.48_1,65_**	**0.038↓**	0.00_1,65_	0.954	**4.87_1,65_**	**0.031** ^a^

^a^ The samples collected >3 m from mountain birches differ between grazing regimes.

### 3.2 Fungal abundance, diversity and community composition

**FIGURE 1:**
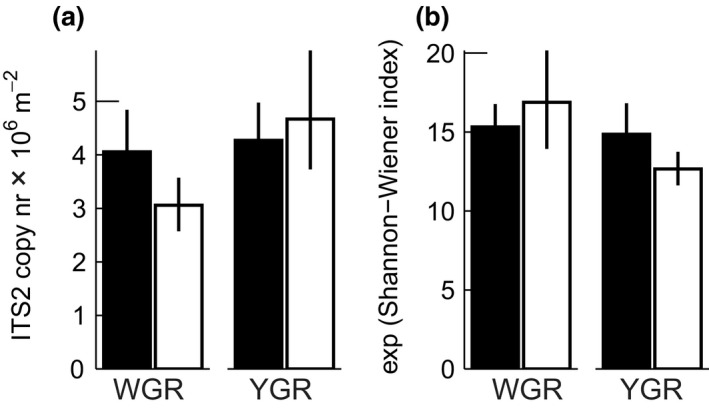
Fungal abundance (a) and effective diversity (b) in the organic soil layer in the vicinity (black bars) and >3 m away from mountain birch trees (white bars) in the winter grazing regime (WGR) and year‐round (YGR) grazing regime. Bars present mean and 95% confidence interval

Fungal abundance in the organic soil layer was higher in the area exposed to year‐round grazing (Figure 1a; Table 1). However, the YGR had lower species richness and evenness (Figure S4b,c; Table 1), resulting in a decreased effective diversity (Figure 1b; Table 1). All diversity indices were indicatively lowest in the YGR >3 m away from birches (Figure 1b; Figure S4b–d), yet the interaction between grazing regime and tree vicinity was significant only for the inverse Simpson diversity index that was higher away from the birches in the WGR than away from the birches in the YGR (Table 1; Figure S4d).

**FIGURE 2:**
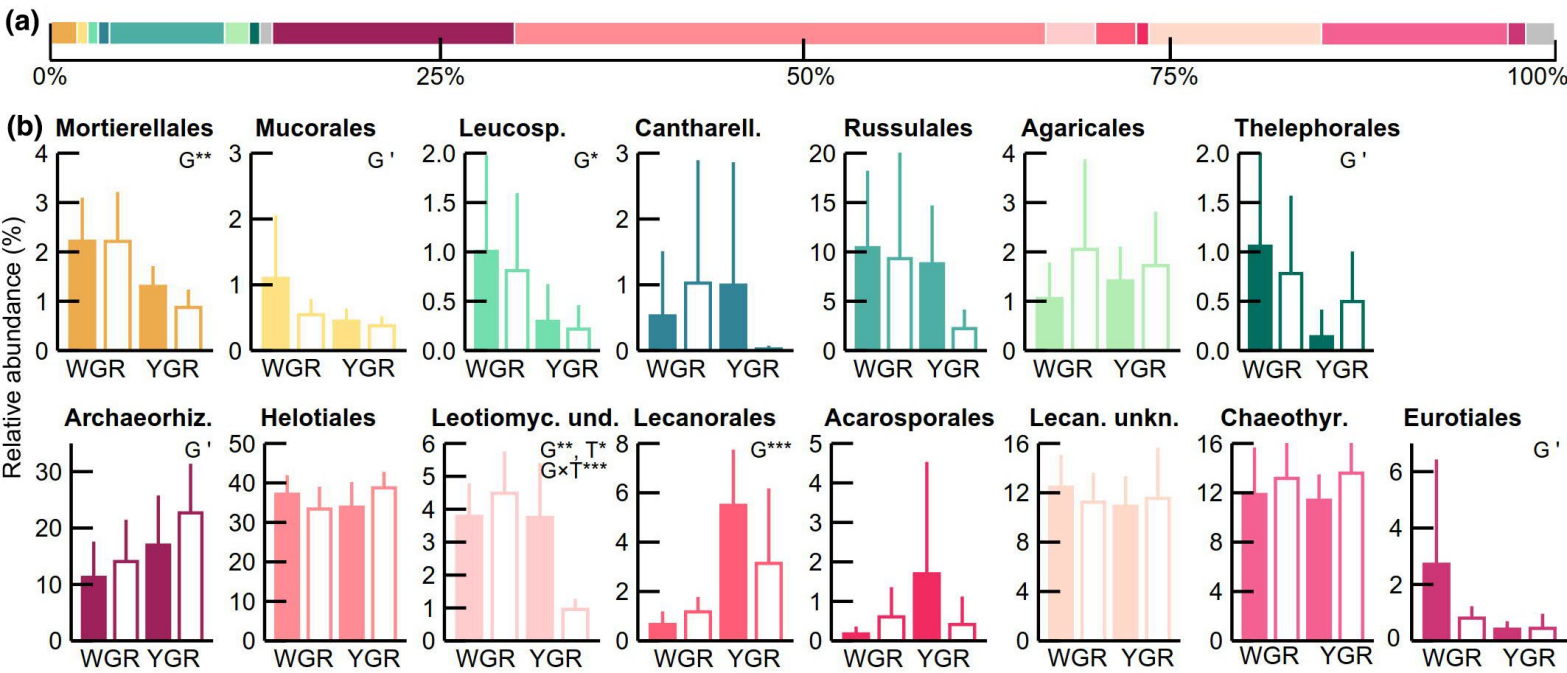
Relative abundance of the most common fungal orders (relative abundance > 0.5%; a), and the response of each order to mountain birch vicinity (filled bars = in close vicinity, and open bars >3 m from mountain birch) and grazing regime (b). Orders are colour‐coded according to Phylum with yellows = Mucoromycota, greens = Basidiomycota and reds = Ascomycota and with light grey parts in (a) indicating the pooled relative abundances of the less common Basidiomycota and Ascomycota orders, in respective order. Bars represent mean ± 95% confidence interval in (b). Significant and close‐to‐significant differences among grazing regimes and tree vicinities are highlighted above the panels (′*p* < 0.1, *p < 0.05, **p < 0.01, ***p < 0.001; see Table S2 for further statistical parameters). Archaeorhiz., Archaeorhizomycetales; Chaetothy, Chaetohyriales; Lecan. und., undefined order in the class Lecanoromycetes; Leotiomyc. und., undefined order in the class Leotiomycetes; Leucosp., Leucosporidiales; WGR, winter grazing regime; YGR, year‐round grazing regime

The grazing regime also affected the taxonomic and functional composition of the fungal community, with grazing impacts evident on phylum, order and species levels (Table S1). The orders Mortierellales, Mucorales, Leucosporidiales, Thelephorales and Eurotiales were significantly or close‐to‐significantly more abundant in the WGR (Table S2; Figure 2). Instead, the phylum Asomycota, the orders Archaeorhizomycetales and Lecanorales and the most abundant species *Pezoloma ericae* were more abundant in the YGR (Figure 2; Figure S5; Table S2). Tree vicinity had little effect on the taxonomic composition, yet tree vicinity interacted with grazing to explain relative abundances of fungal orders and species (Tables S1 and S2; Figure 2).

**FIGURE 3:**
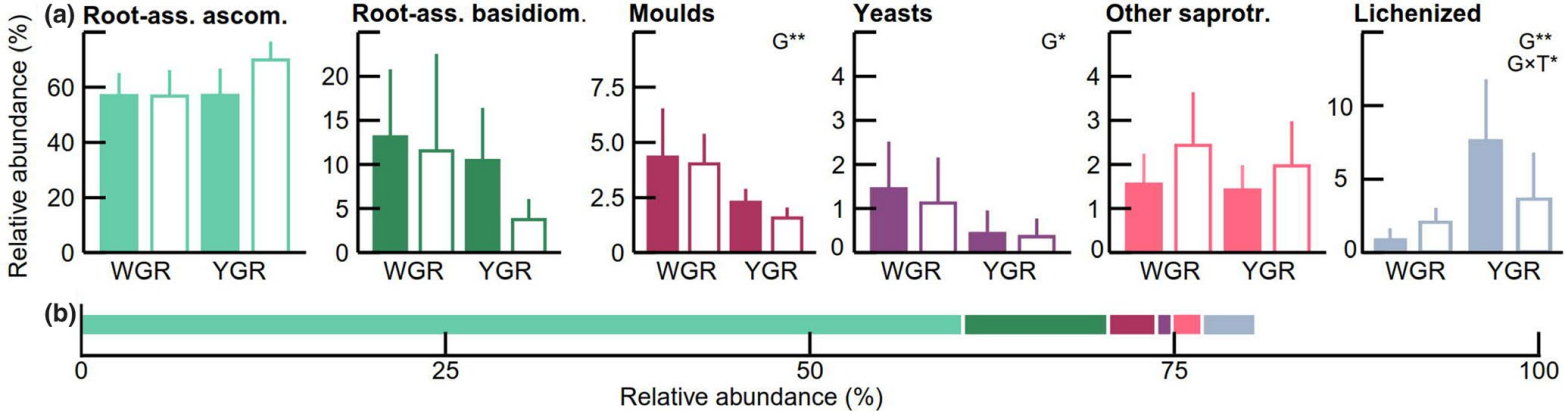
Relative abundances of fungal guilds (mean ± 95% confidence interval) in close vicinity (filled bars) and >3 m away from mountain birches (open bars) in the winter grazing regime (WGR) and year‐round (YGR) grazing regimes (a), and the relative abundance of these guilds across all treatments (b). Significant differences among grazing regimes and tree vicinities are highlighted above the panels (* *p* < 0.05, ** *p* < 0.01, *** *p* < 0.001; see Table S3 for further statistical parameters). Other saprotr., other saprotrophs; Root‐ass. ascom., root‐associated ascomycetes; Root‐ass. basidiom., root‐associated basidiomycetes

For fungal functional guilds, the grazing difference was most evident in moulds, yeasts and lichenized fungi (Figure 3; Table S3). The relative abundances of moulds and yeasts were significantly higher in the WGR than in the YGR (*F*_1,69_ = 8.50, *p* = 0.009 for moulds and *F*_1,69_ = 5.76, *p* = 0.017 for yeasts), whereas lichenized fungi were more abundant in the YGR (F_1,69_ = 13.40, *p* = 0.002; Figure 3). Furthermore, the abundance of lichenized fungi increased away from the trees in the WGR but decreased away from trees in the YGR (Figure 3; Table S3). The abundances of root‐associated guilds were not significantly different between the grazing regimes nor dependant on the tree vicinity (Figure 3; Table S3). Abundances of ErM and EcM fungi strongly reflected the abundances of root‐associated ascomycetes and basidiomycetes, respectively (Figure S6; Figure 3). Most EcM fungi at our site had simple mycelia, with cord‐forming EcM contributing less than 1% of all the fungal reads (Figure S6). The inclusion of mycorrhizal or exploration types did not significantly increase the explanatory power of grazing or tree vicinity in the generalized linear model of the functional guild composition (Table S1).

**FIGURE 4:**
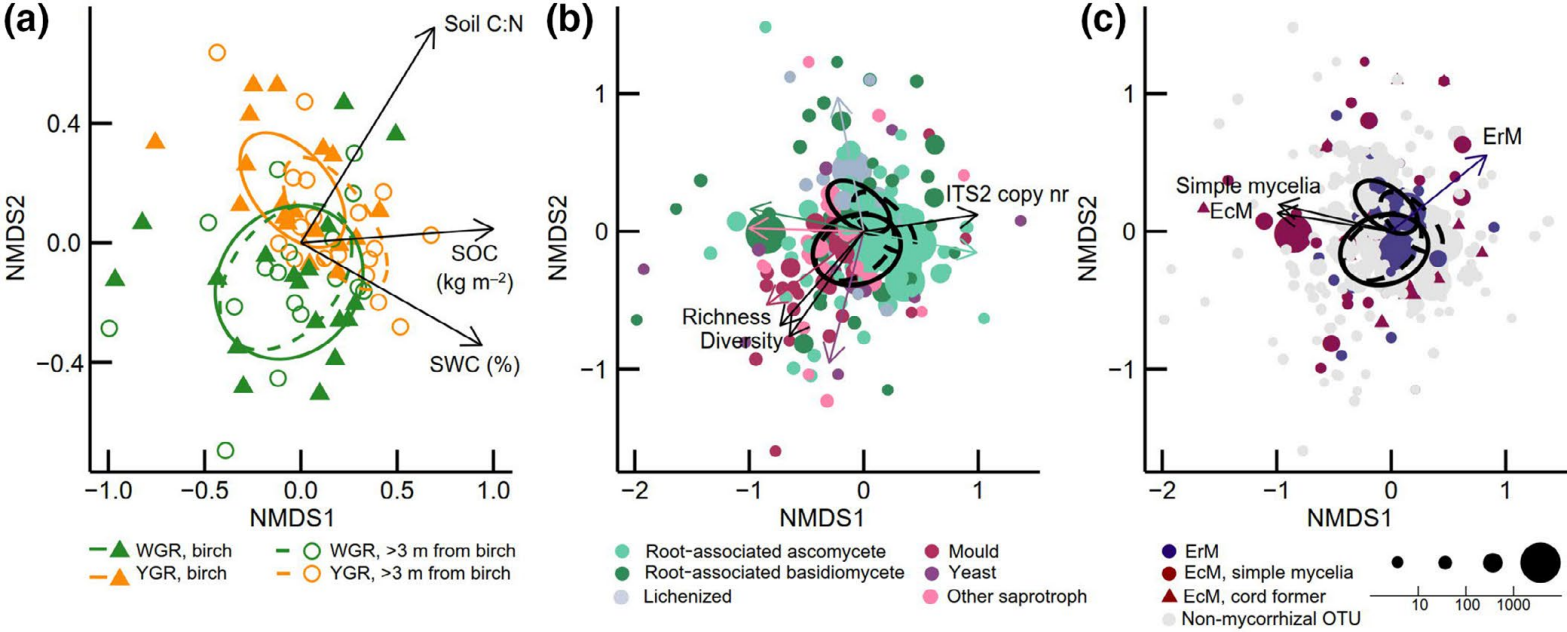
Non‐metric multidimensional scaling of fungal communities in close vicinity (closed symbols) or >3 m from mountain birches (open symbols) in the two grazing regimes (WGR = winter grazing regime, green symbols; YGR = year‐round grazing regime, yellow symbols; a), operational taxonomical units (OTUs) colour‐coded by functional guilds (b) and OTUs colour‐coded by mycorrhizal types (c). The non‐metric multidimensional scaling (NMDS) in based on all OTUs (*n* = 364, stress = 0.240, *r* ^2^ = 0.943), yet, in frame (b), only OTUs with a known function (*n* = 224) are shown. Black vectors present soil C:N ratio (soil C:N), organic soil carbon stocks (SOC kg m^−2^), soil water content (SWC, %), fungal abundance (ITS2 copy number m^−2^), fungal species richness and effective diversity (i.e. the exponent of Shannon–Wiener index). Coloured vectors indicate the directions towards, which the relative abundances of fungal guilds, mycorrhizal types and ectomycorrhizal fungi (EcM) with simple mycelia increase (*r* ^2^ values and significances in Table S5)

In the NMDS, grazing exerted the strongest impact on the community composition (*F*_1,67_ = 2.99, *p* = 0.001; Figure 4). Fungal communities in the YGR aligned with positive NMDS2 axis values in parallel to higher soil C:N ratios, lower OTU richness and diversity (Figure 4a,b) and higher abundance of ErM fungi (Figure 4c). The fungal community in the WGR was characterized by moulds and yeasts (Figure 4a,b **)**, particularly the mould species within *Mortierella* (from the order Mortierellales), *Umbelopsis* (Mucorales) and *Penicillium* (Eurotiales) and the yeast orders Leucoporidiales and Saccharomycetales (Figure S8b; Tables S4 and S5). No impact of grazing or tree vicinity was found on other saprotrophic fungi (Figure 3; Table S3).

Tree vicinity also affected fungal community composition in the NMDS (*F*_1,67_ = 1.62, *p* = 0.020), although this effect was partly masked by its interaction with the grazing regime (*F*_1,67_ = 1.29, *p* = 0.091) indicating that tree vicinity was more important under the YGR (Figure 4a). Fungal communities away from birches in the year‐round grazed area were typically dominated by root‐associated ascomycetes (Figure 4b), particularly belonging to the most abundant orders, Helotiales and Archaeorhizomycetales (Figure S8a; Table S5). Additionally, fungal abundance (ITS2 copies m^−2^) increased away from trees (Figure 4b; Table S5). Fungal communities in the vicinity of trees instead had higher abundances of root‐associated basidiomycetes (Figure 4b), particularly the EcM orders Russulales and Cantharellales (Figure 2; Figure S8a; Table S5).

### 3.3 Associations between fungal community and vegetation DNA

Across all samples, the abundance of *Betula* reads in the soil correlated positively with the relative abundance of guilds associated with deciduous shrubs and trees (i.e. root‐associated basidiomycetes, EcM and the two EcM exploration types, Figure S7b,c; Table S6). Particularly, more EcM were found in plots with more *Betula* reads (*F*_1,69_ = 6.67, *p* = 0.012, *r* ^2^ = 0.09; Figure S7c; Table S6). Both ErM‐associated plant groups, *Vaccinium* sp. and other ericaceous shrubs, correlated positively with root‐associated ascomycetes, while correlating negatively with root‐associated basidiomycetes, EcM and saprotrophic fungi (Figure S7e,h; Table S6). The abundance of lichenized fungi was positively linked to bryophyte abundance (*F*_1,69_ = 10.65, *p* = 0.002, *r* ^2^ = 0.13), whereas saprotrophic fungi correlated negatively with bryophyte abundance (Figure S7k; Table S6).

### 3.4 SOC and its relation to fungal abundance and community composition

**FIGURE 5:**
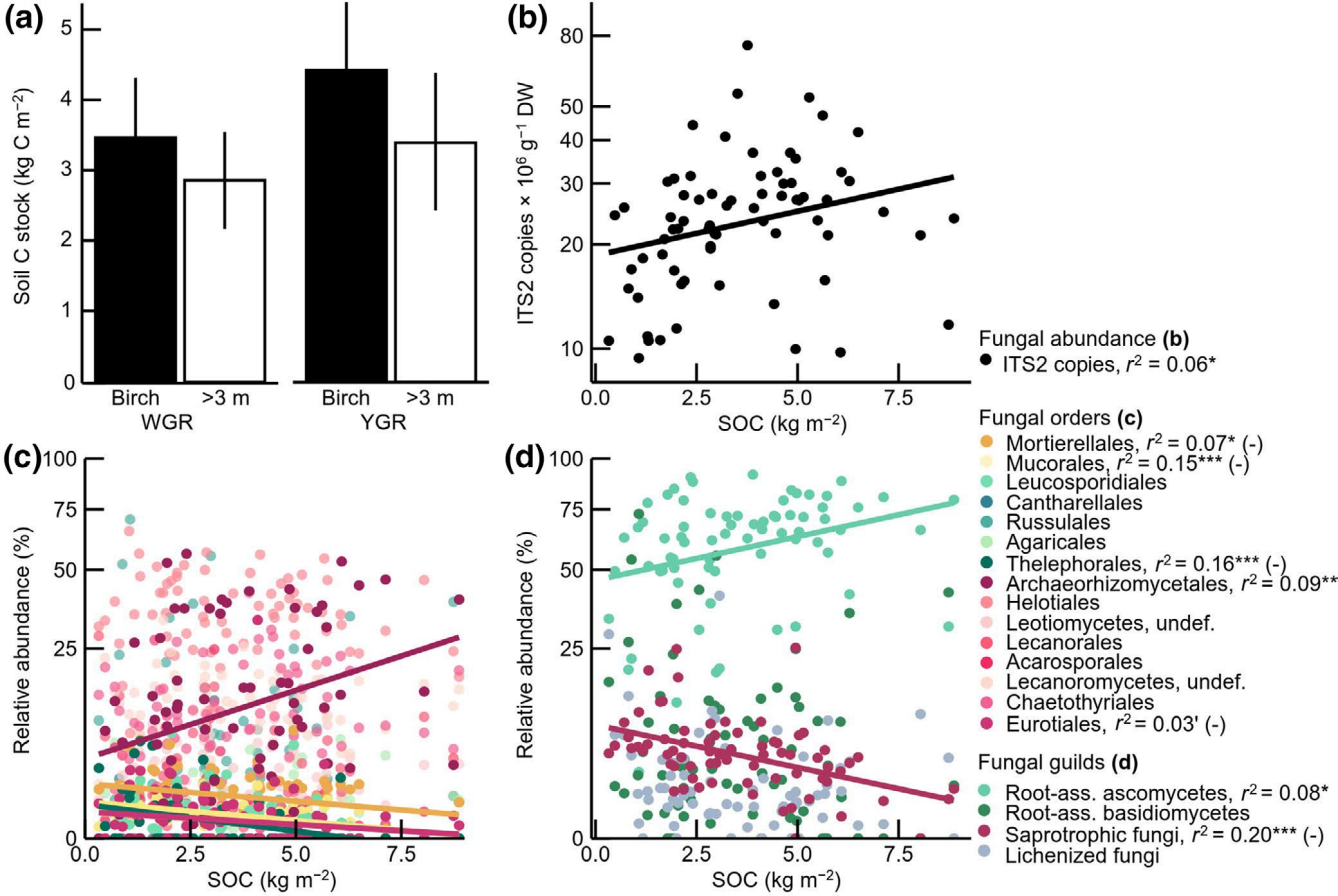
Organic soil carbon stocks (a) and their correlation with fungal abundance (b), fungal orders (c) and functional guilds (d). Significant and close‐to‐significant correlations are indicated with lines (adjusted *r* ^2^‐value and significance of correlation (′*p* ≤ 0.1, *p ≤ 0.05, **p ≤ 0.01, ***p ≤ 0.001) found in the list to the right). In frame (d), moulds, yeasts and other saprotrophs are pooled into one group of saprotrophic fungi. Negative correlations are indicated with a – sign

Organic soil stored on average (±SD) 3.18 ± 1.73 kg C m^−2^ in the WGR and 3.92 ± 2.18 kg C m^−2^ in the YGR (Figure 5a), yet the difference between the grazing regimes was not significant (*F*_1,65_ = 2.55, *p* = 0.115). Instead, closer vicinity to trees tended to increase soil carbon stocks (*F*_1,65_ = 3.15, *p* = 0.081) aligning with the observed pattern in soil nitrogen stocks (Figure S9; Table 1). In the NMDS, SOC increased in the same direction as fungal abundance (Figure 4a,b), and these variables correlated also in the linear model (Figure 5b; Table S7). Furthermore, the same taxonomic groups that aligned with fungal abundance along the NMDS2‐axis (Figure S8) related to soil carbon storage (Figure 5c; Table S7). High abundance of saprotrophic fungi, that is, moulds, yeasts and other saprotrophs, was associated with low SOC stocks (*F*_1,68_ = 17.41, *p* < 0.001, *r* ^2^ = 0.20; Figure 5d), whereas root‐associated ascomycetes were more abundant on plots with high SOC stocks (F_1,68_ = 6.74, *p* = 0.012, r^2^ = 0.08; Figure 5d; Table S7).

When the individual and interactive impacts of grazing and tree vicinity were included in linear models of SOC stocks, fungal orders and functions explained SOC stocks even better (Table S8). For most taxa and guilds, accounting for the slightly higher SOC stocks in the vicinity of mountain birches (Figure 5a), that is, the hummocks formed around trees, the explanatory power of the model increased (Table S8). After including the effect of tree vicinity, also the abundance of root‐associated basidiomycetes was negatively related with SOC stocks (Table S8).

## 4 DISCUSSION

We compared soil fungal communities in two contrasting long‐term reindeer grazing regimes in an alpine treeline ecotone. In one regime, ~70 years of year‐round grazing had hindered mountain birch establishment and led to an open landscape dominated by low stature evergreen dwarf shrubs, while the other regime with only wintertime grazing was characterized by a higher density of mountain birch (Oksanen et al., 1995). We found that the YGR was characterised by higher soil C:N ratio and that the shift in dominant vegetation was accompanied by altered abundance, diversity and community composition of the soil fungi. Although carbon storage in the organic soil layer did not differ significantly between the two grazing regimes, fungal community composition was linked to vegetation composition and organic soil carbon stocks across all experimental plots. This supports the theory that herbivory constraints on deciduous shrub abundance in the tree‐line ecotone may increase soil carbon stocks through the accompanying shifts in vegetation and associated soil fungi (Vowles & Björk, 2019).

### 4.1 Linkages between fungal community composition and soil carbon storage

In line with our first hypothesis and some earlier studies linking reindeer grazing to altered fungal community composition and growth (Santalahti et al., 2018; Vowles et al., 2018), we found that root‐associated ascomycetes, including ErM fungi, were most abundant away from mountain birches in the grazing regime exposed to year‐round reindeer grazing and tightly linked to the abundance of ericaceous dwarf shrubs. In contrast, the abundance of root‐associated basidiomycetes, mostly forming EcM, was linked to the relative abundance of *Betula* DNA in the soil. Moreover, in support of our second hypothesis and observations from boreal forests (Clemmensen et al., 2013, 2015), we show that the abundance of root‐associated ascomycetes correlated positively with soil carbon, whereas root‐associated basidiomycetes correlated negatively with soil carbon when tree vicinity—and thus hummock formation around tree trunks—was accounted for. However, in contrast to our third hypothesis and earlier observations from the treeline (Clemmensen et al., 2021), the negative correlation of EcM fungi with soil carbon stocks could not be linked to EcM species that possess cord‐forming mycelial structures. Instead, all the dominant EcM fungi in the study area, *Russula claroflava*, *Russula decolorans*, *Russula paludosa*, *Pseudotomentella tristis* (Thelephorales), *Elaphomyces muricatus* (Ascomyceta) and species from the order Cantharellales, were characterized by simple mycelia, whereas cord‐forming EcM fungi contributed less than 1% of fungal communities. We suggest this pattern to be linked to the high sensitivity of cord‐forming EcM, such as *Cortinarius*, to disturbances and climatic constraints (Vowles et al., 2018; Clemmensen et al., 2021). Thus, even the smaller disturbances induced by winter time grazing—the digging of soil and vegetation and the loss of an insulating snow layer in the grazing craters—could have benefitted EcM with short‐distance mycelia at the same time as the higher nutrient availability (i.e. low C:N ratio) reduced the demand for traits adapted to the break‐down and long‐distance transport of organically bound nutrients (Bödeker et al., 2014; Kyaschenko et al., 2017; Clemmensen et al., 2021).

Furthermore, in line with our hypotheses, we found that free‐living saprotrophic fungi were more abundant in the WGR and that their abundance correlated negatively with soil carbon storage. These patterns were driven by the moulds Mortierellales, Mucorales, Eurotiales (*Penicillium* sp.) and the yeasts Leucosporidiales and Saccharomycetales. These moulds and yeasts are known for their opportunistic nature and dependence on easily available carbon as their own capacity to decompose complex OM is rather limited (Fernandez & Kennedy, 2018; Lindahl et al., 2010). Thus, the higher abundance of moulds and yeasts in the WGR is likely linked to the increased availability of easily decomposable leaf, root and mycelial litter (McLaren et al., 2017; Parker et al., 2018; Vankoughnett & Grogan, 2016). This is supported by the negative correlations between the abundance of saprotrophic fungi and DNA from plant groups with recalcitrant litters (i.e. bryophytes and the ericaceous species). As saprotroph abundance further correlated positively with EcM abundance, we suggest that they are particularly linked to mycelial biomass turnover of short‐distance EcM fungi (Brabcová et al., 2016; Fernandez & Kennedy, 2018; Lindahl et al., 2010). In contrast to the ErM mycelia with melanised cell walls (Read & Perez‐Moreno, 2003), EcM necromass is less recalcitrant and thus, easier to decompose by moulds and yeasts (Brabcová et al., 2016; Lindahl et al., 2010) and, in later stages of decomposition, also by EcM fungi (Fernandez & Kennedy, 2018). This activity of moulds, yeasts and EcM fungi could prevent carbon and nitrogen from becoming locked up in mycelial necromass (Kyaschenko et al., 2019) allowing a more rapid nutrient cycling and thereby facilitating the nutrient transfer between EcM fungi and their symbiotic trees. Ultimately, the positive feedback loop between plant and fungal communities could contribute to higher soil fertility as suggested by the lower C:N ratio in the WGR.

The soil fungal communities away from the trees in the YGR were highly dominated by two taxa of root‐associated ascomycetes – the well‐characterized ericoid mycorrhizal *P. ericae* and the functionally little understood species within *Archaeorhizomycetes*—that together contributed 41 ± 20% of all fungal reads in these soils. Like the dominant plants in the tundra heaths, *E. hermaphroditum*, *P. caerulea* and *C. vulgaris*, these fungi are likely tolerant to the harsh conditions and to the low nutrient availability that the vegetation contributes to maintaining. Indeed, the plants in ErM‐dominated heaths are characterised by a high content of defence compounds, a high C:N ratio, and in the case of the dominant evergreen shrub *E. hermaphroditum*, even high levels of allelopathic secondary compounds, which retard litter decomposition and nutrient mineralization rates (Tybirk et al., 2000). The recalcitrant necromass of root‐associated ascomycetes may further push tundra heath ecosystems towards lower OM decomposition rates (Clemmensen et al., 2021), not only because of the melanised cell walls of the ErM fungi (Fernandez & Kennedy, 2018) but also because the fungal necromass can form recalcitrant complexes with plant root derived condensed tannins, contributing to nutrients becoming locked up in soil OM (Adamczyk et al., 2019). Ultimately, these feedbacks could contribute to higher rates of soil carbon sequestration (Clemmensen et al., 2021) as also indicated by the positive correlation between the abundance of root‐associated ascomycetes and soil carbon stocks.

### 4.2 Patterns in fungal diversity and abundance and the counterintuitive pattern in lichen abundance

The mountain birch dominated vegetation in the WGR supported higher species richness and effective diversity of the soil fungal community, whereas all diversity indices were indicatively lowest in the heath plots away from the trees in the YGR. These differences are likely linked to the high dominance of *P*. *ericae* and *Archaeorhizomycetes* in the soils away from the trees in the YGR. Yet, the diversity pattern could also indicate a positive response of fungal diversity to an intermediate level of disturbance prevalent in the WGR. In contrast, fungal abundance (as inferred from ITS2 copies) was highest away from the trees in the YGR. Also this pattern is likely linked to the dominant taxa, *P*. *ericae* and *Archaeorhizomycetes*: the combination of the mycelias' high recalcitrancy (Fernandez & Kennedy, 2018) and limited decomposer capacity (Kyaschenko et al., 2017) could explain why higher fungal abundance did not contribute to lower soil carbon stocks (in contrast to Ylänne et al., 2020).

We found the abundance of DNA from lichenized fungi in the organic soil to be higher in the YGR compared with the WGR. This observation was contrary to our expectations, as both satellite images (Cohen et al., 2013; Forbes & Kumpula, 2009) and visual documentation indicate higher cover of particularly reindeer lichens (*Cladonia* spp.) in the WGR than in the YGR (32.1 ± 7.4% in WGR vs. 12.1 ± 3.9% in YGR). As also the lichenized DNA consisted mainly of *Cladonia* species, an important winter forage for reindeer, the results indicate a discrepancy between above‐ and belowground lichen abundance. This difference could derive from biases in the DNA‐based data, such as differences in DNA extraction efficiency or variation in ITS2 copy numbers among species. Yet, given the known vulnerability of lichens to grazing and trampling (Bernes et al., 2015), we suggest that the more intense trampling in the YGR potentially resulted in a higher degree of fragmentation of the above‐ground lichen communities and led to lichen necromass becoming incorporated into the litter and soil layers to a greater degree. The differences between the grazing intensities could further be accentuated by the impact of the environment on the decay rate of lichen necromass (Lang et al., 2009). As the abundance of lichenized fungi was particularly high under the trees in the YGR, where the OM rich hummocks underneath the trees were characterized by mosses from the families *Polytrichum* and *Dicranum*, these could contribute to a more acidic environment, where lichen fractions embedded in the moss layer remain, at least to a certain extent, protected from decay.

### 4.3 Grazing impacts at the landscape level

When interpreting the results of this study at the landscape level, it is important to note that the sampling design largely overestimates the share of mountain birch habitats in the YGR and that the sampling approach did not include fungal communities in the deeper soil layers. Together, these factors could contribute to real differences between the grazing regimes being even larger than seen in this study. Due to differences in rooting depth of these vegetation types, also carbon stocks in deeper roots and soil may differ between the grazing regimes. Yet, the pattern in soil carbon across the landscape is unlikely to merely reflect the pattern in above‐ground biomass and carbon uptake because trees and associated microbial communities also induce decomposition of old and more recalcitrant carbon (Hartley et al., 2012; Parker et al., 2020) resulting in an inverse relationship between the above‐ and belowground carbon stocks in mountain birch forests and tundra heaths (Hartley et al., 2012; Parker et al., 2015; Clemmensen et al., 2021). Our results highlight that such differences between these vegetation types may be induced by long‐term reindeer summer‐time browsing of deciduous trees. Yet, it remains unknown how common it is that grazers drive such vegetation transitions and how generalizable the belowground consequences are. In general, grazer impacts on ecosystems depend on the density, frequency and seasonality of the animal activity. When occurring in high numbers in the snow‐free period, grazers may limit deciduous shrub and tree establishment and growth (Biuw et al., 2014; Bråthen et al., 2017; Post & Pedersen, 2008). Yet, not all deciduous shrub species respond to herbivory similarly. For example, the resinous nature of the North‐American dwarf birches (*B*. *nana* ssp. *exilis* and *Betula glandulosa*) and alders (*Alnus* sp.) may, to certain extent, protect these from grazing (Christie et al., 2015). Furthermore, grazers' preferential browsing of deciduous shrubs may provide a competitive advantage for either evergreen dwarf shrubs (Sundqvist et al., 2019; Vowles et al., 2017; Ylänne et al., 2015) or grasses and forbs (Egelkraut et al., 2018; Olofsson et al., 2004; Post & Pedersen, 2008), which likely associate with disparate grazer controls on belowground communities and soil carbon sequestration. Further, certain deciduous shrub species form symbiotic associations with ErM or arbuscular mycorrhizal fungi, and thus the belowground consequences of a grazer‐induced vegetation shift from deciduous to evergreen shrubs may diverge, depending on the mycorrhizal type of the dominant shrubs (sensu Soudzilovskaia et al., 2019). Thus, attributes linked to grazers, shrubs, mycorrhizas and other fungi all act in concert to determine the belowground consequences induced by grazing.

### 4.4 Increasing importance of grazing in the rapidly warming Arctic

It is well established that vertebrate herbivores, when occurring in high numbers and at a particular time, may exert negative effects on the abundance of deciduous shrubs and trees (Christie et al., 2015; Olofsson & Post, 2018). As vertebrate herbivory mainly prevents the establishment of new trees (Biuw et al., 2014; Kumpula et al., 2011), it only induces vegetation shifts from forest to open landscapes when accompanied by other disturbances that induce tree mortality, such as outbreaks of invertebrate herbivores (Biuw et al., 2014) or pathogens (Olofsson et al., 2011). However, ample evidence from across the arctic tundra has shown that vertebrate herbivores prevent climate‐induced advancement of deciduous shrubs (Kaarlejärvi et al., 2015; Post & Pedersen, 2008; Zamin & Grogan, 2013) and keep tree‐lines lower than their altitudinal limits (Cairns & Moen, 2004; Oksanen et al., 1995; Van Bogaert et al., 2011). In this context, it is becoming clear that vertebrate herbivory alone may be decisive for vegetation trajectories in a warming tundra (Bråthen et al., 2017; Christie et al., 2015; Olofsson & Post, 2018). This highlights the need to incorporate current grazing ranges for improved predictions of tundra vegetation development (Yu et al., 2017). Yet, it also emphasizes the need to predict the consequences of altered grazing patterns induced by societal and environmental changes, such as the dramatic declines in wild reindeer herds (Russell et al., 2018).

The northward and altitudinal expansion of shrubs and trees is occurring across Arctic and subarctic regions (Myers‐Smith et al., 2019; Tape et al., 2012; Terskaia et al., 2020) and is expected to continue (Bjorkman et al., 2020). Although most observations of shrub expansion derive from riparian areas and tundra meadows (Bjorkman et al., 2020; Bråthen et al., 2017; Post & Pedersen, 2008), areas dominated by evergreen dwarf shrubs have also been reported to be encroached by tall deciduous shrubs in recent years (Terskaia et al., 2020). Such expansions are notable in scale as tundra heaths are the most abundant vegetation type in the Arctic, covering 13.6% of the vegetated land surface (classified as erect dwarf shrub tundra in the Circumpolar Arctic Vegetation map; CAVM Team, 2003). By combating the encroachment of deciduous shrubs and trees in these areas, both large and smaller herbivores could be decisive for shrub and tree ranges in a warmer climate. Here we suggest that when controlling the ranges of mountain birch forests and tundra heaths, herbivory has the potential to alter belowground fungal communities and soil carbon sequestration at the treeline ecotone. As these two vegetation types associate with different above‐ and belowground feedbacks linked to the dominant vegetation (Wardle et al., 2004) and to the soil fungal community (Read et al., 2004; Vowles & Björk, 2019; Clemmensen et al., 2021), herbivory could control a tipping‐point between the two vegetation trajectories and be decisive for the belowground consequences of climate change.

## 5 CONCLUSIONS

Here we showed that ~70 years of different reindeer grazing regimes at the treeline ecotone shifted vegetation, soil fungal communities and soil carbon sequestration in disparate directions. We found that mountain birch woodlands experiencing only winter grazing hosted a fungal community characterized by short‐distance EcM fungi and free‐living saprotrophs and were linked to high soil fertility. In contrast, year‐round grazing by reindeer promoted a more open tundra heath vegetation associated with a fungal community dominated by a few root‐associated ascomycete species, whose abundance correlated with higher organic soil carbon stocks. These findings show that when controlling the abundance of deciduous shrubs and trees, vertebrate herbivores may have far‐reaching consequences on key below‐ground processes and, ultimately, on soil carbon sequestration. This supports earlier suggestions about the potential to use reindeer management (Bråthen et al., 2017) and even reintroductions of large grazers (Olofsson & Post, 2018) to reduce some of the unwanted effects of a warmer climate, such as shrub encroachment and the associated loss of soil carbon. Our study also highlights that past, current and future grazing patterns are important to include when predicting the belowground consequences of the ongoing climatic and environmental changes across the Arctic tundra.

## AUTHOR CONTRIBUTION

Henni Ylänne, Daniel B. Metcalfe and Karina E. Clemmensen designed the experiment and Daniel B. Metcalfe financed the study. Henni Ylänne and Rieke L. Madsen collected and pre‐processed the soil samples. Rieke L. Madsen and Carles Castaño conducted the molecular analyses and bioinformatics. Henni Ylänne designed and performed the statistical analyses and led the writing of the manuscript to which all other authors commented.

## Supporting information

Supplementary Material

Supplementary Material

## Data Availability

All data that support the findings of this study is openly available. Sequence data are archived at NCBI's Sequence Read Archive with the name ‘Soil fungal communities in the alpine treeline ecotone under contrasting reindeer grazing regimes’ (PRJNA730715; www.ncbi.nlm.nih.gov/sra/PRJNA730715). Sample data and scripts are available in Dryad and Zenodo with the name ‘Data from Reindeer control over subarctic treeline alters soil fungal communities with potential consequences for soil carbon storage’ (https://doi.org/10.5061/dryad.mkkwh70zs).

## References

[gcb15722-bib-0001] Abarenkov, K. , Henrik Nilsson, R. , Larsson, K.‐H. , Alexander, I. J. , Eberhardt, U. , Erland, S. , Høiland, K. , Kjøller, R. , Larsson, E. , Pennanen, T. , Sen, R. , Taylor, A. F. S. , Tedersoo, L. , Ursing, B. M. , Vrålstad, T. , Liimatainen, K. , Peintner, U. , & Kõljalg, U. (2010). The UNITE database for molecular identification of fungi – Recent updates and future perspectives. New Phytologist, 186(2), 281–285. 10.1111/j.1469-8137.2009.03160.x 20409185

[gcb15722-bib-0002] Abarenkov, K. , Somervuo, P. , Nilsson, R. H. , Kirk, P. M. , Huotari, T. , Abrego, N. , & Ovaskainen, O. (2018). Protax‐fungi: A web‐based tool for probabilistic taxonomic placement of fungal internal transcribed spacer sequences. New Phytologist, 220(2), 517–525. 10.1111/nph.15301 30035303

[gcb15722-bib-0003] Adamczyk, B. , Sietiö, O. M. , Biasi, C. , & Heinonsalo, J. (2019). Interaction between tannins and fungal necromass stabilizes fungal residues in boreal forest soils. New Phytologist, 223(1), 16–21. 10.1111/nph.15729 30721536

[gcb15722-bib-0004] Agerer, R. (2006). Fungal relationships and structural identity of their ectomycorrhizae. Mycological Progress, 5(2), 67–107. 10.1007/s11557-006-0505-x

[gcb15722-bib-0005] Barrio, I. C. , Bueno, C. G. , Gartzia, M. , Soininen, E. M. , Christie, K. S. , Speed, J. D. M. , Ravolainen, V. T. , Forbes, B. C. , Gauthier, G. , Horstkotte, T. , Hoset, K. S. , Høye, T. T. , Jónsdóttir, I. S. , Lévesque, E. , Mörsdorf, M. A. , Olofsson, J. , Wookey, P. A. , & Hik, D. S. (2016). Biotic interactions mediate patterns of herbivore diversity in the Arctic. Global Ecology and Biogeography, 25(9), 1108–1118. 10.1111/geb.12470

[gcb15722-bib-0006] Bernes, C. , Bråthen, K. A. , Forbes, B. C. , Hofgaard, A. , Moen, J. , & Speed, J. D. (2015). What are the impacts of reindeer/caribou (*Rangifer tarandus* L.) on arctic and alpine vegetation? A systematic review protocol. Environmental Evidence, 2(1), 6. 10.1186/2047-2382-2-6

[gcb15722-bib-0007] Biuw, M. , Jepsen, J. U. , Cohen, J. , Ahonen, S. H. , Tejesvi, M. , Aikio, S. , Wäli, P. R. , Vindstad, O. P. L. , Markkola, A. , Niemelä, P. , & Ims, R. A. (2014). Long‐term impacts of contrasting management of large ungulates in the Arctic tundra‐forest ecotone: Ecosystem structure and climate feedback. Ecosystems, 17(5), 890–905. 10.1007/s10021-014-9767-3

[gcb15722-bib-0008] Bjorkman, A. D. , Garcıá Criado, M. , Myers‐Smith, I. H. , Ravolainen, V. , Jónsdóttir, I. S. , Westergaard, K. B. , & Normand, S. (2020). Status and trends in Arctic vegetation: Evidence from experimental warming and long‐term monitoring. Ambio, 49, 678–692. 10.1007/s13280-019-01161-6 30929249 PMC6989703

[gcb15722-bib-0009] Bödeker, I. T. M. , Clemmensen, K. E. , de Boer, W. , Martin, F. , Olson, Å. , & Lindahl, B. D. (2014). Ectomycorrhizal *Cortinarius* species participate in enzymatic oxidation of humus in northern forest ecosystems. New Phytologist, 203(1), 245–256. 10.1111/nph.12791 24725281

[gcb15722-bib-0010] Brabcová, V. , Nováková, M. , Davidová, A. , & Baldrian, P. (2016). Dead fungal mycelium in forest soil represents a decomposition hotspot and a habitat for a specific microbial community. New Phytologist, 210(4), 1369–1381. 10.1111/nph.13849 26832073

[gcb15722-bib-0011] Bråthen, K. A. , Ravolainen, V. T. , Stien, A. , Tveraa, T. , & Ims, R. A. (2017). Rangifer management controls a climate‐sensitive tundra state transition. Ecological Applications, 27(8), 2416–2427. 10.1002/eap.1618 28871616

[gcb15722-bib-0012] CAFF (Conservation of Arctic Flora and Fauna) . CAFF map no. 28 – Distribution of wild reindeer, caribou, and domestic reindeer in the Arctic (2001). Retrieved from http://library.arcticportal.org/id/eprint/1358

[gcb15722-bib-0013] Cairns, D. M. , & Moen, J. (2004). Herbivory influences tree lines. Journal of Ecology, 92(6), 1019–1024. 10.1111/j.1365-2745.2004.00945.x

[gcb15722-bib-0014] Castaño, C. , Berlin, A. , Brandström Durling, M. , Ihrmark, K. , Lindahl, B. D. , Stenlid, J. , Clemmensen, K. E. , & Olson, Å. (2020). Optimized metabarcoding with Pacific biosciences enables semi‐quantitative analysis of fungal communities. New Phytologist, 228(3), 1149–1158. 10.1111/nph.16731 32531109

[gcb15722-bib-0015] CAVM Team . (2003). Circumpolar Arctic vegetation map. Scale 1:7,500,000. Conservation of Arctic Flora and Fauna (CAFF) map no. 1. U.S. Fish and Wildlife Service.

[gcb15722-bib-0016] Christie, K. S. , Bryant, J. P. , Gough, L. , Ravolainen, V. T. , Ruess, R. W. , & Tape, K. D. (2015). The role of vertebrate herbivores in regulating shrub expansion in the Arctic: A synthesis. BioScience, 65(12), 1123–1133. 10.1093/biosci/biv137

[gcb15722-bib-0017] Clemmensen, K. E. , Bahr, A. , Ovaskainen, O. , Dahlberg, A. , Ekblad, A. , Wallander, H. , Stenlid, J. , Finlay, R. D. , Wardle, D. A. , & Lindahl, B. D. (2013). Roots and associated fungi drive long‐term carbon sequestration in boreal forest. Science, 339(6127), 1615–1618. 10.1126/science.1231923 23539604

[gcb15722-bib-0018] Clemmensen, K. E. , Durling, M. B. , Michelsen, A. , Hallin, S. , Finlay, R. D. , & Lindahl, B. D. (2021). A tipping point in carbon storage when forest expands into tundra is related to mycorrhizal recycling of nitrogen. Ecology Letters, 24(6), 1193–1204. 10.1111/ele.13735 33754469

[gcb15722-bib-0019] Clemmensen, K. E. , Finlay, R. D. , Dahlberg, A. , Stenlid, J. , Wardle, D. A. , & Lindahl, B. D. (2015). Carbon sequestration is related to mycorrhizal fungal community shifts during long‐term succession in boreal forests. New Phytologist, 205(4), 1525–1536. 10.1111/nph.13208 25494880

[gcb15722-bib-0020] Clemmensen, K. E. , Ihrmark, K. , Durling, M. B. , & Lindahl, B. D. (2016). Sample preparation for fungal community analysis by high‐throughput sequencing of barcode amplicons. In F. Martin & S. Uroz (Eds.), Methods in molecular biology (pp. 61–88). Springer Science and Business Media. 10.1007/978-1-4939-3369-3_4 26791497

[gcb15722-bib-0021] Cohen, J. , Pulliainen, J. , Ménard, C. B. , Johansen, B. , Oksanen, L. , Luojus, K. , & Ikonen, J. (2013). Effect of reindeer grazing on snowmelt, albedo and energy balance based on satellite data analyses. Remote Sensing of Environment, 135, 107–117. 10.1016/j.rse.2013.03.029

[gcb15722-bib-0022] Cornwell, W. K. , Cornelissen, J. H. C. , Amatangelo, K. , Dorrepaal, E. , Eviner, V. T. , Godoy, O. , Hobbie, S. E. , Hoorens, B. , Kurokawa, H. , Pérez‐Harguindeguy, N. , Quested, H. M. , Santiago, L. S. , Wardle, D. A. , Wright, I. J. , Aerts, R. , Allison, S. D. , van Bodegom, P. , Brovkin, V. , Chatain, A. , … Westoby, M. (2008). Plant species traits are the predominant control on litter decomposition rates within biomes worldwide. Ecology Letters, 11(10), 1065–1071. 10.1111/j.1461-0248.2008.01219.x 18627410

[gcb15722-bib-0023] Dahlgren, J. , Oksanen, L. , Oksanen, T. , Olofsson, J. , Hambäck, P. A. , & Lindgren, Å. (2009). Plant defences to no avail? Responses of plants of varying edibility to food web manipulations in a low arctic scrubland. Evolutionary Ecology Research, 11(8), 1189–1203.

[gcb15722-bib-0024] Edgar, R. C. (2010). Search and clustering orders of magnitude faster than BLAST. Bioinformatics, 26(19), 2460–2461. 10.1093/bioinformatics/btq461 20709691

[gcb15722-bib-0025] Egelkraut, D. , Aronsson, K. Å. , Allard, A. , Åkerholm, M. , Stark, S. , & Olofsson, J. (2018). Multiple feedbacks contribute to a centennial legacy of reindeer on tundra vegetation. Ecosystems, 21(8), 1545–1563. 10.1007/s10021-018-0239-z

[gcb15722-bib-0026] Fernandez, C. W. , & Kennedy, P. G. (2018). Melanization of mycorrhizal fungal necromass structures microbial decomposer communities. Journal of Ecology, 106(2), 468–479. 10.1111/1365-2745.12920

[gcb15722-bib-0027] Forbes, B. C. , & Kumpula, T. (2009). The ecological role and geography of reindeer (*Rangifer tarandus*) in northern Eurasia. Geography Compass, 3(4), 1356–1380. 10.1111/j.1749-8198.2009.00250.x

[gcb15722-bib-0028] Hartley, I. P. , Garnett, M. , Sommerkorn, M. , Hopkins, D. W. , Fletcher, B. J. , Sloan, V. L. , & Wookey, P. A. (2012). A potential loss of carbon associated with greater plant growth in the European Arctic. Nature Climate Change, 2(12), 875–879. 10.1038/nclimate1575

[gcb15722-bib-0029] Ihrmark, K. , Bödeker, I. T. M. , Cruz‐Martinez, K. , Friberg, H. , Kubartova, A. , Schenck, J. , Strid, Y. , Stenlid, J. , Brandström‐Durling, M. , Clemmensen, K. E. , & Lindahl, B. D. (2012). New primers to amplify the fungal ITS2 region – Evaluation by 454‐sequencing of artificial and natural communities. FEMS Microbiology Ecology, 82(3), 666–677. 10.1111/j.1574-6941.2012.01437.x 22738186

[gcb15722-bib-0030] Jepsen, J. U. , Biuw, M. , Ims, R. A. , Kapari, L. , Schott, T. , Vindstad, O. P. L. , & Hagen, S. B. (2013). Ecosystem impacts of a range expanding forest defoliator at the forest‐tundra ecotone. Ecosystems, 16(4), 561–575. 10.1007/s10021-012-9629-9

[gcb15722-bib-0031] Jost, L. (2006). Entropy and diversity. Oikos, 113(2), 363–375. 10.1111/j.2006.0030-1299.14714.x

[gcb15722-bib-0032] Kaarlejärvi, E. , Hoset, K. S. , & Olofsson, J. (2015). Mammalian herbivores confer resilience of Arctic shrub‐dominated ecosystems to changing climate. Global Change Biology, 21(9), 3379–3388. 10.1111/gcb.12970 25967156

[gcb15722-bib-0033] Kitti, H. , Forbes, B. C. , & Oksanen, J. (2009). Long‐ and short‐term effects of reindeer grazing on tundra wetland vegetation. Polar Biology, 32(2), 253–261. 10.1007/s00300-008-0526-9

[gcb15722-bib-0034] Kumpula, J. , Stark, S. , & Holand, Ø. (2011). Seasonal grazing effects by semi‐domesticated reindeer on subarctic mountain birch forests. Polar Biology, 34(3), 441–453. 10.1007/s00300-010-0899-4

[gcb15722-bib-0035] Kyaschenko, J. , Clemmensen, K. E. , Hagenbo, A. , Karltun, E. , & Lindahl, B. D. (2017). Shift in fungal communities and associated enzyme activities along an age gradient of managed *Pinus sylvestris* stands. The ISME Journal, 11(4), 863–874. 10.1038/ismej.2016.184 28085155 PMC5364365

[gcb15722-bib-0036] Kyaschenko, J. , Ovaskainen, O. , Ekblad, A. , Hagenbo, A. , Karltun, E. , Clemmensen, K. E. , & Lindahl, B. D. (2019). Soil fertility in boreal forest relates to root‐driven nitrogen retention and carbon sequestration in the mor layer. New Phytologist, 221(3), 1492–1502. 10.1111/nph.15454 30281792

[gcb15722-bib-0037] Lang, S. I. , Cornelissen, J. H. C. , Klahn, T. , Van Logtestijn, R. S. P. , Broekman, R. , Schweikert, W. , & Aerts, R. (2009). An experimental comparison of chemical traits and litter decomposition rates in a diverse range of subarctic bryophyte, lichen and vascular plant species. Journal of Ecology, 97(5), 886–900. 10.1111/j.1365-2745.2009.01538.x

[gcb15722-bib-0038] Lenth, R. V. (2016). Least‐squares means: The R package lsmeans. Journal of Statistical Software, 69(1), 1–33. 10.18637/jss.v069.i01

[gcb15722-bib-0039] Lindahl, B. D. , de Boer, W. , & Finlay, R. D. (2010). Disruption of root carbon transport into forest humus stimulates fungal opportunists at the expense of mycorrhizal fungi. The ISME Journal, 4, 872–881. 10.1038/ismej.2010.19 20220789

[gcb15722-bib-0040] Lindahl, B. D. , Nilsson, R. H. , Tedersoo, L. , Abarenkov, K. , Carlsen, T. , Kjøller, R. , Kõljalg, U. , Pennanen, T. , Rosendahl, S. , Stenlid, J. , & Kauserud, H. (2013). Fungal community analysis by high‐throughput sequencing of amplified markers – A user’s guide. New Phytologist, 199(1), 288–299. 10.1111/nph.12243 23534863 PMC3712477

[gcb15722-bib-0041] Mazerolle, M. J. (2019). AICcmodavg: Model selection and multimodel inference based on (Q)AIC(c). R package version 2.2‐2. Retrieved from https://cran.r‐project.org/web/packages/AICcmodavg/AICcmodavg.pdf

[gcb15722-bib-0042] McLaren, J. R. , Buckeridge, K. M. , van de Weg, M. J. , Shaver, G. R. , Schimel, J. P. , & Gough, L. (2017). Shrub encroachment in Arctic tundra: *Betula nana* effects on above‐ and belowground litter decomposition. Ecology, 98(5), 1361–1376. 10.1002/ecy.1790 28263375

[gcb15722-bib-0043] McMurdie, P. J. , & Holmes, S. (2013). Phyloseq: An R package for reproducible interactive analysis and graphics of microbiome census data. PLoS One, 8(4), e61217. 10.1371/journal.pone.0061217 23630581 PMC3632530

[gcb15722-bib-0044] Myers‐Smith, I. H. , Grabowski, M. M. , Thomas, H. J. D. , Angers‐Blondin, S. , Daskalova, G. N. , Bjorkman, A. D. , Cunliffe, A. M. , Assmann, J. J. , Boyle, J. S. , McLeod, E. , McLeod, S. , Joe, R. , Lennie, P. , Arey, D. , Gordon, R. R. , & Eckert, C. D. (2019). Eighteen years of ecological monitoring reveals multiple lines of evidence for tundra vegetation change. Ecological Monographs, 89(2), e01351. 10.1002/ecm.1351

[gcb15722-bib-0045] Oksanen, J. , Blanchet, F. G. , Friendly, M. , Kindt, R. , Legendre, P. , McGlinn, D. , Minchin, P. R. , O'Hara, R. B. , Simpson, G. L. , Peter Solymos, M. , Stevens, H. H. , Szoecs, E. & Wagner, H. (2019). vegan: Community ecology package. R package version 2.5‐2. Cran R.

[gcb15722-bib-0046] Oksanen, L. , Moen, J. , & Helle, T. (1995). Timberline Patterns in northemmost Fennoscandia. Acta Botanica Fennica, 153, 93–105.

[gcb15722-bib-0047] Olofsson, J. , Ericson, L. , Torp, M. , Stark, S. , & Baxter, R. (2011). Carbon balance of Arctic tundra under increased snow cover mediated by a plant pathogen. Nature Climate Change, 1(4), 220–223. 10.1038/nclimate1142

[gcb15722-bib-0048] Olofsson, J. , & Post, E. (2018). Effects of large herbivores on tundra vegetation in a changing climate, and implications for rewilding. Philosophical Transactions of the Royal Society B: Biological Sciences, 373(1761), 20170437. 10.1098/rstb.2017.0437 PMC6231078 30348880

[gcb15722-bib-0049] Olofsson, J. , Stark, S. , & Oksanen, L. (2004). Reindeer infuence on ecosystem processes in the tundra. Oikos, 2, 386–396. 10.1111/j.0030-1299.2004.13048.x

[gcb15722-bib-0050] Parker, T. C. , Clemmensen, K. E. , Friggens, N. L. , Hartley, I. P. , Johnson, D. , Lindahl, B. D. , Olofsson, J. , Siewert, M. B. , Street, L. E. , Subke, J.‐A. , & Wookey, P. A. (2020). Rhizosphere allocation by canopy‐forming species dominates soil CO_2_ efflux in a subarctic landscape. New Phytologist, 227(6), 1818–1830. 10.1111/nph.16573 32248524

[gcb15722-bib-0051] Parker, T. C. , Sanderman, J. , Holden, R. D. , Blume‐Werry, G. , Sjögersten, S. , Large, D. , Castro‐Díaz, M. , Street, L. E. , Subke, J.‐A. , & Wookey, P. A. (2018). Exploring drivers of litter decomposition in a greening Arctic: Results from a transplant experiment across a treeline. Ecology, 99(10), 2284–2294. 10.1002/ecy.2442 29981157 PMC6849570

[gcb15722-bib-0052] Parker, T. C. , Subke, J. A. , & Wookey, P. A. (2015). Rapid carbon turnover beneath shrub and tree vegetation is associated with low soil carbon stocks at a subarctic treeline. Global Change Biology, 21(5), 2070–2081. 10.1111/gcb.12793 25367088 PMC4657486

[gcb15722-bib-0053] Pinheiro, J. , Bates, D. , DebRoy, S. , & Sarkar, D. , & R Core Team . (2014). nlme: Linear and nonlinear mixed effects models. R package version 3.1‐118. Retrieved from http://cran.r‐project.org/package=nlme

[gcb15722-bib-0054] Post, E. , & Pedersen, C. (2008). Opposing plant community responses to warming with and without herbivores. Proceedings of the National Academy of Sciences of the United States of America, 105(34), 12353–12358. 10.1073/pnas.0802421105 18719116 PMC2527915

[gcb15722-bib-0055] R Core Team . (2020). R: A language and environment for statistical computing. R Foundation for Statistical Computing. Retrieved from https://www.r‐project.org/

[gcb15722-bib-0056] Read, D. J. , Leake, J. R. , & Perez‐Moreno, J. (2004). Mycorrhizal fungi as drivers of ecosystem processes in heathland and boreal forest biomes. Canadian Journal of Botany, 82, 1243–1263. 10.1139/b05-912

[gcb15722-bib-0057] Read, D. J. , & Perez‐Moreno, J. (2003). Mycorrhizas and nutrient cycling in ecosystems – A journey towards relevance? New Phytologist, 157(3), 475–492. 10.1046/j.1469-8137.2003.00704.x 33873410

[gcb15722-bib-0058] Rheubottom, S. I. , Barrio, I. C. , Kozlov, M. V. , Alatalo, J. M. , Andersson, T. , Asmus, A. L. , Baubin, C. , Brearley, F. Q. , Egelkraut, D. D. , Ehrich, D. , Gauthier, G. , Jónsdóttir, I. S. , Konieczka, S. , Lévesque, E. , Olofsson, J. , Prevéy, J. S. , Slevan‐Tremblay, G. , Sokolov, A. , Sokolova, N. , … Hik, D. S. (2019). Hiding in the background: Community‐level patterns in invertebrate herbivory across the tundra biome. Polar Biology, 42(10), 1881–1897. 10.1007/s00300-019-02568-3

[gcb15722-bib-0059] Russell, D. E. , Gunn, A. , & Kutz, S. (2018). Migratory tundra caribou and wild reindeer. In Arctic report card 2018. Retrieved from https://www.arctic.noaa.gov/Report‐Card

[gcb15722-bib-0060] Santalahti, M. , Sun, H. , Sietiö, O.‐M. , Köster, K. , Berninger, F. , Laurila, T. , Pumpanen, J. , & Heinonsalo, J. (2018). Reindeer grazing alter soil fungal community structure and litter decomposition related enzyme activities in boreal coniferous forests in Finnish Lapland. Applied Soil Ecology, 132(September), 74–82. 10.1016/j.apsoil.2018.08.013

[gcb15722-bib-0061] Schmitz, O. J. , Wilmers, C. C. , Leroux, S. J. , Doughty, C. E. , Atwood, T. B. , Galetti, M. , Davies, A. B. , & Goetz, S. J. (2018). Animals and the zoogeochemistry of the carbon cycle. Science, 362(6419), eaar3213. 10.1126/science.aar3213 30523083

[gcb15722-bib-0062] Soudzilovskaia, N. A. , van Bodegom, P. M. , Terrer, C. , Zelfde, M. V. , McCallum, I. , Luke McCormack, M. , Fisher, J. B. , Brundrett, M. C. , de Sá, N. C. , & Tedersoo, L. (2019). Global mycorrhizal plant distribution linked to terrestrial carbon stocks. Nature Communications, 10, 5077. 10.1038/s41467-019-13019-2 PMC6838125 31700000

[gcb15722-bib-0063] Stark, S. , & Väisänen, M. (2014). Insensitivity of soil microbial activity to temporal variation in soil N in subarctic tundra: Evidence from responses to large migratory grazers. Ecosystems, 17(5), 906–917. 10.1007/s10021-014-9768-2

[gcb15722-bib-0064] Sterkenburg, E. , Clemmensen, K. E. , Ekblad, A. , Finlay, R. D. , & Lindahl, B. D. (2018). Contrasting effects of ectomycorrhizal fungi on early and late stage decomposition in a boreal forest. The ISME Journal, 12(9), 2187–2197. 10.1038/s41396-018-0181-2 29880913 PMC6092328

[gcb15722-bib-0065] Sundqvist, M. K. , Moen, J. , Björk, R. G. , Vowles, T. , Kytöviita, M. , Parsons, M. A. , & Olofsson, J. (2019). Experimental evidence of the long‐term effects of reindeer on Arctic vegetation greenness and species richness at a larger landscape scale. Journal of Ecology, 107(6), 2724–2736. 10.1111/1365-2745.13201

[gcb15722-bib-0066] Tape, K. D. , Hallinger, M. , Welker, J. M. , & Ruess, R. W. (2012). Landscape heterogeneity of shrub expansion in Arctic Alaska. Ecosystems, 15(5), 711–724. 10.1007/s10021-012-9540-4

[gcb15722-bib-0067] Tedersoo, L. , Tooming‐Klunderud, A. , & Anslan, S. (2018). PacBio metabarcoding of fungi and other eukaryotes: Errors, biases and perspectives. New Phytologist, 217(3), 1370–1385. 10.1111/nph.14776 28906012

[gcb15722-bib-0068] Terskaia, A. , Dial, R. J. , & Sullivan, P. F. (2020). Pathways of tundra encroachment by trees and tall shrubs in the western Brooks Range of Alaska. Ecography, 43(5), 769–778. 10.1111/ecog.05015

[gcb15722-bib-0069] Tybirk, K. , Nilsson, M.‐C. , Michelsen, A. , Kristensen, H. L. , Shevtsova, A. , Tune Strandberg, M. , Johansson, M. , Nielsen, K. E. , Riis‐Nielsen, T. , Strandberg, B. , & Johnsen, I. (2000). Nordic empetrum dominated ecosystems: Function and susceptibility to environmental changes. Ambio, 29(2), 90–97. 10.1579/0044-7447-29.2.90

[gcb15722-bib-0070] Van Bogaert, R. , Haneca, K. , Hoogesteger, J. , Jonasson, C. , De Dapper, M. , & Callaghan, T. V. (2011). A century of tree line changes in sub‐Arctic Sweden shows local and regional variability and only a minor influence of 20th century climate warming. Journal of Biogeography, 38(5), 907–921. 10.1111/j.1365-2699.2010.02453.x

[gcb15722-bib-0071] van der Wal, R. (2006). Do herbivores cause habitat degradation or vegetation state transition? Evidence from the tundra. Oikos, 114(1), 177–186. 10.1111/j.2006.0030-1299.14264.x

[gcb15722-bib-0072] Vankoughnett, M. R. , & Grogan, P. (2016). Plant production and nitrogen accumulation above‐ and belowground in low and tall birch tundra communities: The influence of snow and litter. Plant and Soil, 408(1–2), 195–210. 10.1007/s11104-016-2921-2

[gcb15722-bib-0073] Vowles, T. , & Björk, R. G. (2019). Implications of evergreen shrub expansion in the Arctic. Journal of Ecology, 107(2), 650–655. 10.1111/1365-2745.13081

[gcb15722-bib-0074] Vowles, T. , Gunnarsson, B. , Molau, U. , Hickler, T. , Klemedtsson, L. , & Bj, R. G. (2017). Expansion of deciduous tall shrubs but not evergreen dwarf shrubs inhibited by reindeer in Scandes mountain range. Journal of Ecology, 105(6), 1547–1561. 10.1111/1365-2745.12753 29200500 PMC5697633

[gcb15722-bib-0075] Vowles, T. , Lindwall, F. , Ekblad, A. , Bahram, M. , Furneaux, B. R. , Ryberg, M. , & Björk, R. G. (2018). Complex effects of mammalian grazing on extramatrical mycelial biomass in the Scandes forest‐tundra ecotone. Ecology and Evolution, 8(2), 1019–1030. 10.1002/ece3.3657 29375775 PMC5773333

[gcb15722-bib-0076] Wang, Y. , Naumann, U. , Wright, S. T. , & Warton, D. I. (2012). *Mvabund* – An R package for model‐based analysis of multivariate abundance data. Methods in Ecology and Evolution, 3, 461–474. 10.1111/j.2041-210X.2012.00190.x

[gcb15722-bib-0077] Wardle, D. A. , Bardgett, R. D. , Klironomos, J. N. , Setälä, H. , van der Putten, W. H. , & Wall, D. H. (2004). Ecological linkages between aboveground and belowground biota. Science, 304(5677), 1629–1633. 10.1126/science.1094875 15192218

[gcb15722-bib-0078] White, T. J. , Bruns, S. , Lee, S. , & Taylor, J. (1990). Amplification and direct sequencing of fungal ribosomal RNA genes for phylogenetics. In M. A. Innis , D. H. Gelfand , J. J. Sninsky , & T. J. White (Eds.), PCR protocols: A guide to methods and applications (pp. 315–322). Academic Press. 10.1016/b978-0-12-372180-8.50042-1

[gcb15722-bib-0079] Wickham, H. (2016). ggplot2: Elegant graphics for data analysis. Springer‐Verlag. Retrieved from https://ggplot2.tidyverse.org

[gcb15722-bib-0080] Ylänne, H. , Kaarlejärvi, E. , Väisänen, M. , Männistö, M. K. , Ahonen, S. H. K. , Olofsson, J. , & Stark, S. (2020). Removal of grazers alters the response of tundra soil carbon to warming and enhanced nitrogen availability. Ecological Monographs, 90(1), e01396. 10.1002/ecm.1396

[gcb15722-bib-0081] Ylänne, H. , Stark, S. , & Tolvanen, A. (2015). Vegetation shift from deciduous to evergreen dwarf shrubs in response to selective herbivory offsets carbon losses: Evidence from 19 years of warming and simulated herbivory in the subarctic tundra. Global Change Biology, 21(10), 3696–3711. 10.1111/gcb.12964 25950664

[gcb15722-bib-0082] Yu, Q. , Epstein, H. E. , Engstrom, R. , & Walker, D. A. (2017). Circumpolar arctic tundra biomass and productivity dynamics in response to projected climate change and herbivory. Global Change Biology, 23(9), 3895–3907. 10.1111/gcb.13632 28276177

[gcb15722-bib-0083] Zamin, T. J. , & Grogan, P. (2013). Caribou exclusion during a population low increases deciduous and evergreen shrub species biomass and nitrogen pools in low Arctic tundra. Journal of Ecology, 101(3), 671–683. 10.1111/1365-2745.12082

[gcb15722-bib-0084] Zhang, W. , Jansson, C. , Miller, P. A. , Smith, B. , & Samuelsson, P. (2014). Biogeophysical feedbacks enhance the arctic terrestrial carbon sink in regional earth system dynamics. Biogeosciences, 11(19), 5503–5519. 10.5194/bg-11-5503-2014

