# Supplementary material for: Reindeer control over subarctic treeline alters soil fungal communities with potential consequences for soil carbon storage
Source: Glob Chang Biol. 2021 Jun 14;27(18):4254–68. doi: 10.1111/gcb.15722 (PMC13420783; doi:10.1111/gcb.15722)
Supplement: Supplementary file 1 — Supplementary Material [file GCB-27--s001.pdf]

Supplementary Figures and Tables for "Reindeer control over subarctic treeline alters soil fungal communities with potential consequences for soil carbon storage" by Henni Ylänne, Rieke L. Madsen, Carles Castaño, Daniel B. Metcalfe and Karina E. Clemmensen

The following supplementary figures and tables are found in this file:

Fig. S1 The study area in Finland with year-round presence of reindeer (**a**), and in Norway with reindeer present only in winter (**b**).

Fig. S2 The study-set up, showing the placement of original coordinates in one of the three blocks (**a**) and the collection of soil samples from each original coordinate, where the closest birch and the closest area with at least 3 meters distance to the closest birch were selected for sampling (**b**).

Fig. S3 Soil moisture (**a**), C stocks (**b**), N stocks (**c**) and soil C:N ratio (**d**) in close vicinity and more than 3 m away from mountain birches in the winter and year-round grazing regimes.

Fig. S4 Fungal abundance as expressed for g soil (**a**), fungal species richness (**b**), Pielous' evenness index (**c**), and inverse Simpson diversity index (**d**) in close vicinity and more than 3 m away from mountain birches in the winter and year-round grazing regimes.

Fig S5. Relative abundances of known genera and species within the fungal orders.

Fig. S6 Relative abundances of ericoid mycorrhizal (ErM, **a**), ectomycorrhizal (EcM, **b**), and cord-forming EcM (**c**) fungi in close vicinity and more than 3 m away from mountain birches in the winter and year-round grazing regimes.

Fig. S7 Indicative abundance of *Betula* sp. (**a**), *Vaccinium* sp. (**d**), other ericaceous species (**g**) and bryophytes (**j**) in close vicinity and more than 3 m away from mountain birches in the winter and year-round grazing regimes and their correlations with fungal functional guilds (**b, e, h, k**) and mycorrhizal types (**c**, **f**, **i**).

Fig. S8 Fungal orders within root-associated ascomycetes and basidiomycetes (**a**) and moulds, yeasts and other saprotrophic and litter associated fungi (**b**) in the NMDS of the community.

Fig. S9 Soil nitrogen stocks in close vicinity and more than 3 m away from mountain birches in the winter and year-round grazing regimes (**a**) and their correlation with soil carbon stocks (**b**).

Table S1 Results of the multivariate generalized linear model on the impact of grazing regime, tree vicinity and their interaction on the different taxonomic levels and the functional guilds.

Table S2 Univariate test results on the impact of grazing regime, tree vicinity, and their interaction on the most abundant fungal phyla, orders and species in the organic soil.

Table S3 Univariate test results on the impact of grazing regime, tree vicinity, and their interaction on the most abundant fungal orders and species within the functional guilds.

Table S4 Univariate test results on the impact of grazing regime, tree vicinity, and their interaction on the relative abundance of most abundant fungal orders and species within the functional guilds.

Table S5 Explanatory value of vectors in the NMDS ordination (*envfit* results)

Table S6. Correlations between the captured ITS2 reads of vegetation and fungal guilds, mycorrhizal types and EcM exploration types.

Table S7 Results of Akaikes' information criteria test on how well fungal abundance, taxonomy (i.e. fungal orders complemented with significantly correlating genera and species) and guilds explain soil organic carbon stocks.

Table S8 Summary of best explanatory value models on how fungal abundance, orders, and functional guilds explain SOC when the individual and/or interacting effect of grazing regime and/or tree vicinity is included.

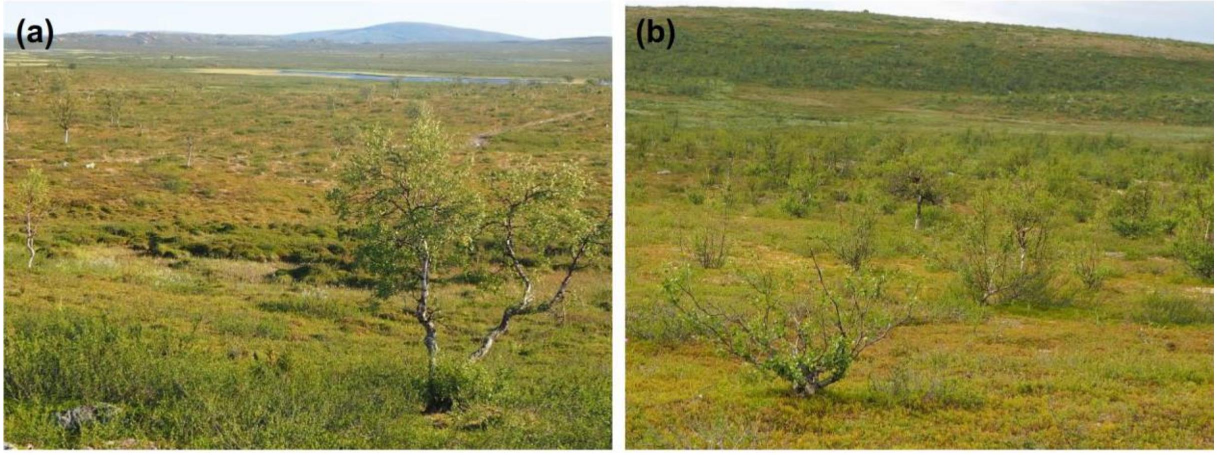

Fig. S1 The study area in Finland with year-round presence of reindeer (**a**), and in Norway with reindeer present only in winter (**b**).

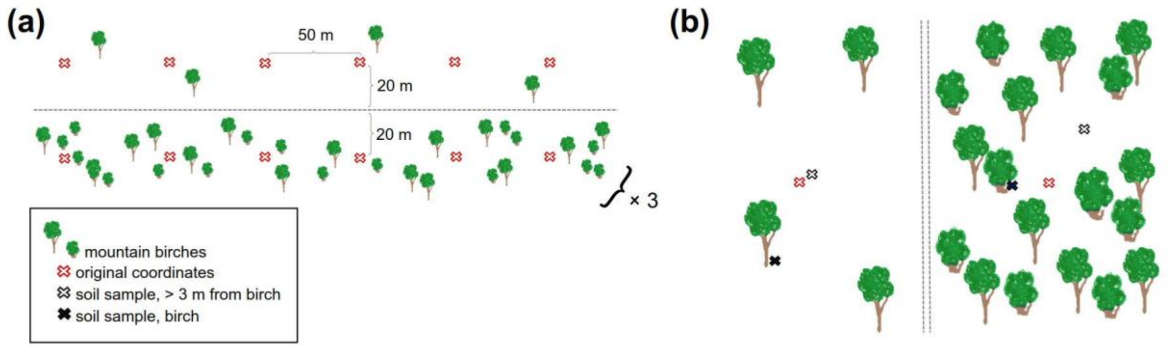

Fig. S2 The study-set up, showing the placement of original coordinates along the fence separating the two grazing regimes in one of the three blocks (**a**) and the collection of soil samples from each original coordinate, where the closest mountain birch tree and the closest area with at least 3 meters distance to the closest mountain birch tree were selected for sampling (**b**). From the original coordinates, we moved on average 3 m to the closest heath-dominated area (max distance 20 m) and on average 33 m to the closest mountain birch (max distance 150 m).

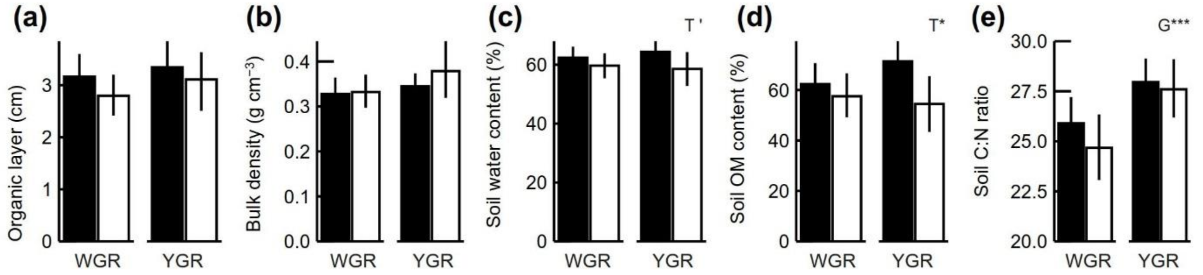

Fig. S3 Organic layer depth (**a**), bulk density (**b**), water content (**c**), organic matter content (**d**) and C:N ratio (**e**) in close vicinity (black bars) and more than 3 m away (white bars) from mountain birches in the winter (WGR) and year-round (YGR) grazing regimes. Values present mean ± 95% confidence interval. The asterisks above the panels present significant and close-to-significant grazing (G) and tree (T) effects.

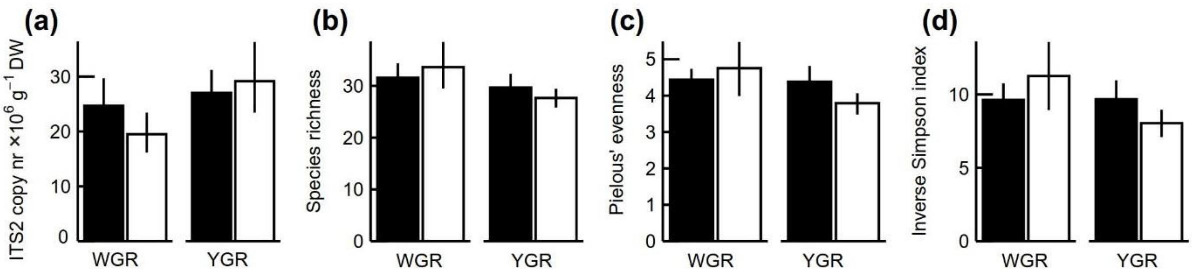

Fig. S4 Fungal abundance for g DW (**a**), fungal species richness (**b**), Pielous' evenness index (**c**), and inverse Simpson diversity index (**d**) in close vicinity (black bars) and more than 3 m away (white bars) from mountain birches under in the winter (WGR) and year-round (YGR) grazing regimes. Figures show mean and 95% confidence interval.

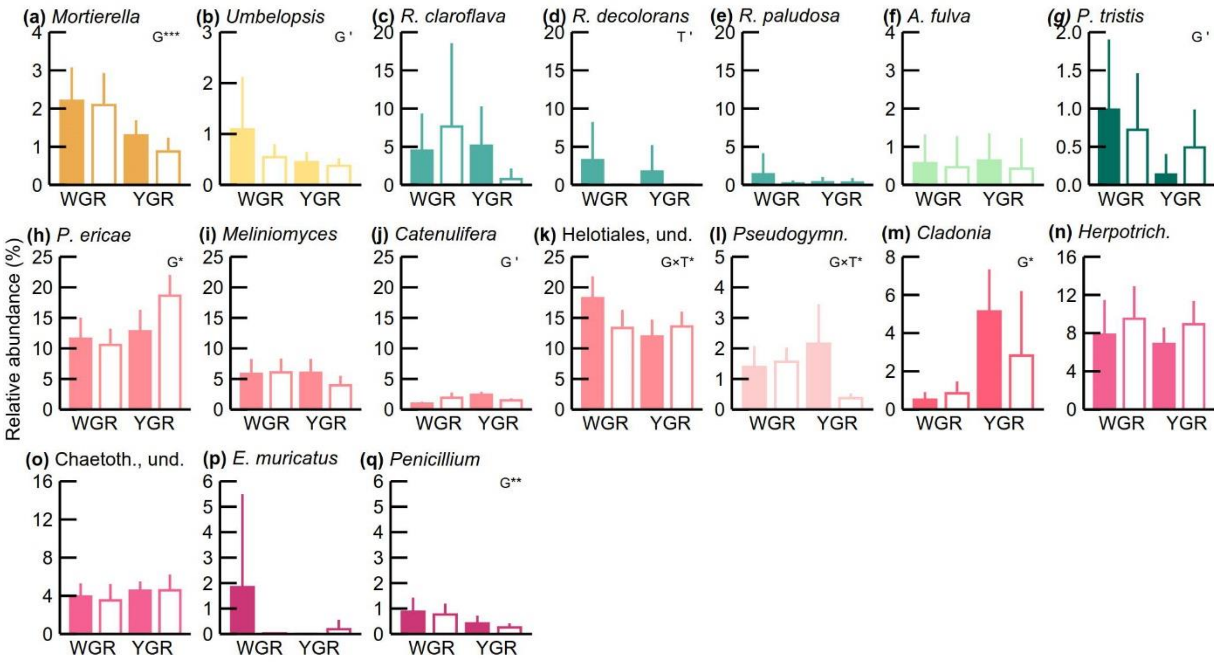

Fig. S5 Relative abundances of known genera or species within the fungal orders in close vicinity (filled bars) and more than 3 m away (white bars) from mountain birches under in the winter (WGR) and year-round (YGR) grazing regimes. Full names of genera and species: *Mortierella* (belongs to the order Mortierellales, **a**), *Umbelopsis* (Mucorales, **b**), *Russula claroflava* (Russulales, **c**), *Russula decolorans* (Russulales, **d**), *Russula paludosa* (Russulales, **e**), *Amanita fulva* (Agaricales, **f**), *Pseudotomentella tristis* (Thelephorales, **g**), *Pezoloma ericae* (Helotiales, **h**), Meliniomyces (Helotiales, **i**), *Catenulifera* (Helotiales, **j**), unidentified fungi within the order Helotiales (**k**), *Pseudogymnoascus* (Leotiomycetes, undef. order, l), *Cladonia* (Lecanorales, **m**), *Herpotrichiellaceae* (Chaetothyriales, **n**), unidentified fungi within the order Chaetorhyriales (**o**), *Elaphomyces muricatus* (Eurotiales, **p**) and *Penicillium* (Eurotiales, **q**). Color-coding and scales based on orders shown in Fig. 2. Significant and close-to-significant impacts of grazing regime (G) or tree vicinity (T) are indicated with asterisks, where *** $p < 0.001$, ** $p < 0.01$, * $p < 0.05$ and ′ $p < 0$ .1 (full list of univariate results in Table S2).

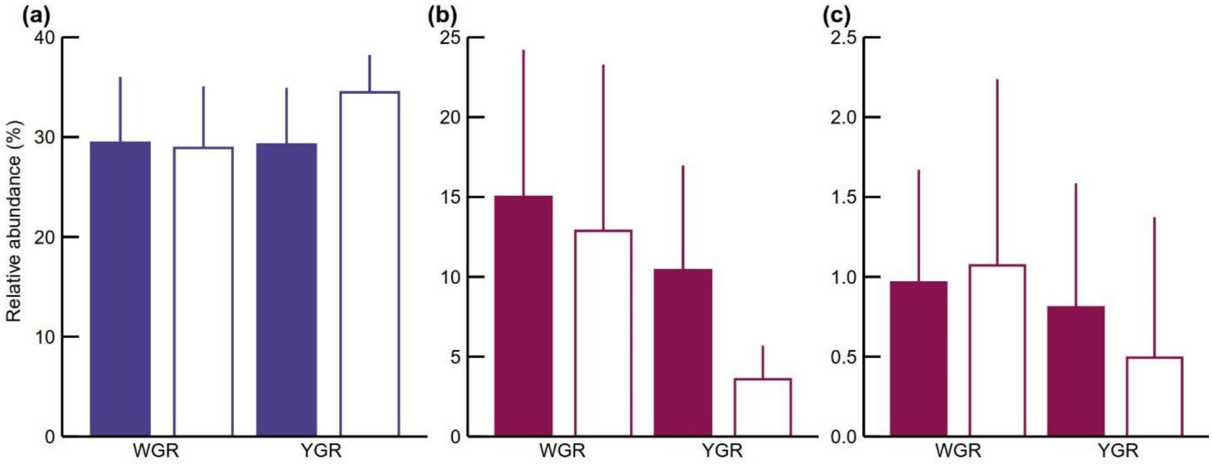

Fig. S6 Relative abundances (mean and 95% confidence interval) of ericoid mycorrhiza (ErM, **a**), ectomycorrhiza (EcM, **b**), and cord-forming EcM (**c**) in close vicinity (filled bars) and more than 3 m away (white bars) from mountain birches in the winter (WGR) and year-round (YGR) grazing regimes.

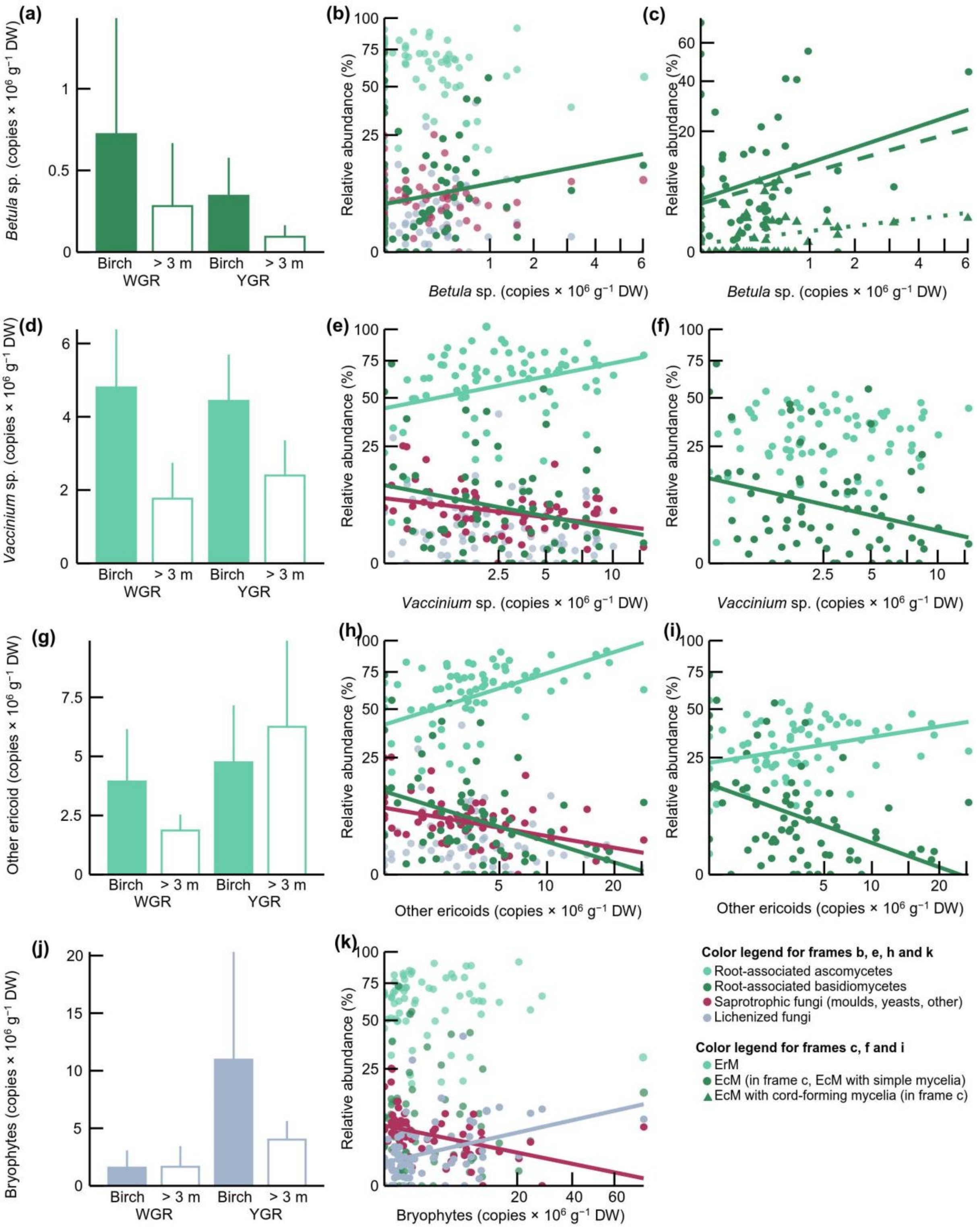

Fig. S7 Indicative abundance (mean and 95% confidence interval) of *Betula* sp. (**a**), *Vaccinium* sp. (**d**), other ericaceous species (i.e. *Empetrum nigrum* spp. *hermaprhoditum*, *Phyllodoce caerulea* and *Calluna vulgaris*) (**g**) and bryophytes (**j**) in close vicinity (filled bars) and more than 3 m away (white bars) from mountain birches in the winter (WGR) and year-round (YGR) grazing regimes. Frames (**b**), (**e**), (**h**) and (**k**) indicate correlations of the plant groups with fungal guilds and frames (**c**), (**f**) and (**i**) correlations with ericoid mycorrhizal (ErM) and

ectomycorrhizal (EcM) fungi. Significant correlation are shown with lines (full list in Table S6). In frame (**c**), correlations are shown separately for all EcM (solid line), EcM with simple mycelia (dashed line) and cord-forming EcM (dotted line). The abundance of mycorrhizal types was not linked to bryophyte abundance (Table S6) and is therefore not visualized here.

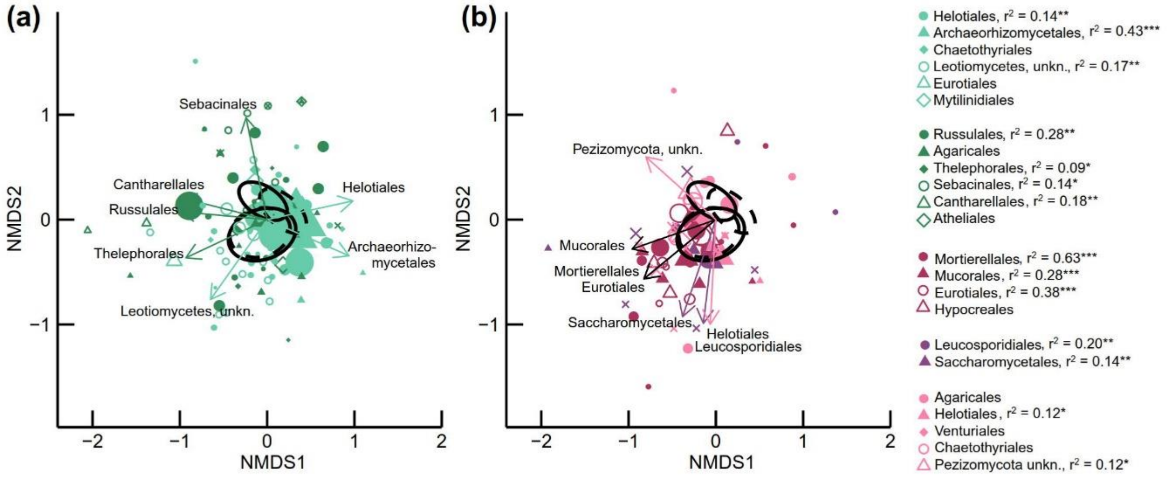

Fig. S8 Fungal orders within root-associated ascomycetes and basidiomycetes (**a**, in light and dark green, respectively) and moulds, yeasts and other saprotrophic fungi (**b**, in red, violet and light pink, respectively) in the NMDS of the community (Fig. 4b). OTUs from less abundant orders (relative abundance < 0.1%) are marked with ×. Vectors, r^2 -value and significance of correlation (* $p \leq 0.05$, ** $p \leq 0.01$, *** $p \leq 0.001$) are only shown for orders aligning with the NMDS (fit of the remaining orders in Table S4). In frame (b), the arrows for Mortierellales and Eurotiales align. Black ellipses represent the treatments (Fig. 4a), where the lower ellipses enclose samples collected from the winter grazing regime, and the upper ones samples collected from the year-round gazing regime. Samples close to trees are surrounded with even lines, and samples > 3 m from trees are surrounded by dashed lines.

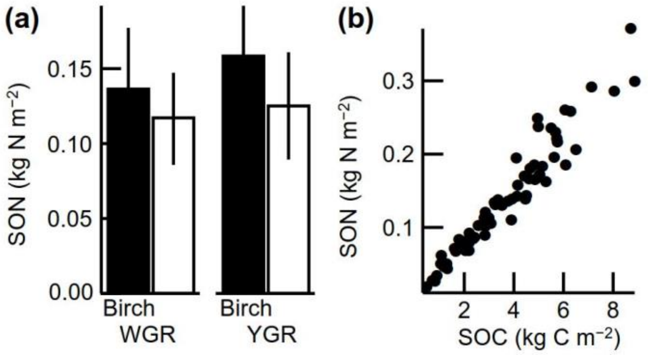

Fig. S9 Soil nitrogen stocks in close vicinity (black bars) and more than 3 m away (white bars) from mountain birch trees in the winter (WGR) and year-round (YGR) grazing regimes (**a**) and their correlation with soil carbon stocks (**b**). Bars present mean and 95% confidence interval.

Table S1 Results of the multivariate generalized linear model on the impact of grazing regime, tree vicinity and their interaction on the different taxonomic levels and the functional guilds.

	**Nr of groups**	**Grazing**		**Tree**		**Grazing × Tree**	
		**$Dev_{1,69}$**	***p***	**$Dev_{1,68}$**	***p***	**$Dev_{1,67}$**	***p***
Phyla	4	**15.42**	**0.016**	4.61	0.214	3.61	0.269
Subphyla	6	5.09	0.398	4.68	0.394	8.31	0.159
Class	10	8.64	0.431	10.26	0.294	8.93	0.376
Order	15	**47.30**	**0.001**	13.87	0.288	**25.60**	**0.027**
Species	21	**48.38**	**0.001**	16.82	0.344	**32.13**	**0.023**
Functional guild (incl. unknown)	7	**32.17**	**0.001**	5.86	0.335	8.88	0.152
Functional guild	6	**31.22**	**0.001**	5.63	0.248	8.46	0.105
+ mycorrhizal type (incl. unknown)	8	**33.16**	**0.002**	6.52	0.343	8.53	0.192
+ mycorrhizal type	6	**28.84**	**0.015**	3.58	0.738	9.59	0.304
+ exploration type (incl. unknown)	8	**35.43**	**0.002**	5.47	0.451	10.76	0.104
+ exploration type	7	**31.54**	**0.001**	5.46	0.339	8.00	0.141
Orders within functional guilds	15	**49.02**	**0.001**	8.79	0.570	18.51	0.094
Species within functional guilds	44	**85.63**	**0.001**	29.87	0.573	**50.03**	0.174

First test on functional guild include the root-associated ascomycetes, root-associated basidiomycetes, moulds, yeasts, other saprotrophic and lichenized fungi as well as fungi with unknown function. As none of the model parameters explained the variation in unknown fungi, the generalized linear model was repeated without the fungi with unknown function. Subsequently, the root-associated guilds were divided into ErM, EcM, other root-associated ascomycetes, and other root-associated basidiomycetes. As none of the model parameters explained the variation of the root-associated groups with unknown mycorrhizal type, the generalized linear model was repeated with only the known mycorrhizal types. Thirdly, ectomycorrhizal fungi were separated into the following exploration types: cord-forming EcM, EcM with simple mycelia, and EcM with unknown exploration type. As none of the model parameters explained the variation in EcM with unknown exploration type, the generalized linear model was repeated with only the known exploration types.

As mycorrhizal and exploration types were explored by replacing root-associated guilds with the two mycorrhizal types, and subsequently, by replacing EcM fungi with the two exploration types, the grazing and tree impact of these models should only be interpreted as a change from the previous.

Univariate results for phyla, order and species shown in Table S2, for functional guilds in Table S3, and for orders and species within the functional guilds in Table S4.

Table S2 Univariate test results on the impact of grazing regime, tree vicinity, and their interaction on the relative abundance of most abundant fungal phyla, orders and species in the organic soil. Arrows indicate increase (↑) or decrease (↓) towards the year-round grazing regime or closer vicinity to mountain birch, directions of significant grazing and tree interactions are noted in footnotes.

Taxa	Rel . abu.	Grazing $Dev_{1,69}$	Grazing *p*	Tree $Dev_{1,68}$	Tree *p*	Grazing × Tree $Dev_{1,67}$	Grazing × Tree *p*
**Ascomycota**	85.8	**2.65**	**0.050**↑	0.74	0.333	0.50	0.383
Acarosporales	0.7	0.95	0.381	0.01	0.924	1.67	0.219
Archaeorhizomycetales	16.3	1.61	0.069↑	0.53	0.293	0.01	0.894
Chaetothyriales	12.5	0.00	0.998	0.94	0.346	0.06	0.819
Herpotrichiellaceae	8.3	0.19	0.594	1.21	0.200	0.03	0.820
Chaetothyriales	4.2	0.53	0.276	0.03	0.770	0.05	0.726
Eurotiales	1.1	7.91	0.054↓	1.23	0.364	1.84	0.287
Penicillium	0.6	**4.09**	**0.007**↓	0.51	0.376	0.16	0.635
Elaphomyces muricatus	0.5	0.54	0.565	0.05	0.745	3.11	0.163
Helotiales	35.8	0.11	0.739	0.04	0.851	2.05	0.143
Helotiales, undefined spec.	14.3	3.55	0.054↓	0.59	0.460	**3.90**	**0.044a**
Pezoloma ericae	13.4	**4.60**	**0.011**↑	0.84	0.264	2.20	0.073
Meliniomyces	5.5	0.55	0.376	0.66	0.359	0.99	0.275
Catenulifera	1.7	1.68	0.073↑	0.18	0.592	**6.35**	**0.003b**
Lecanorales	2.6	**15.09**	**0.001**↑	0.00	1.000	2.43	0.090
Cladonia, undefined species	2.3	**15.80**	**0.001**↑	0.02	0.882	1.90	0.131
Lecanoromycetes, undef. order	11.5	0.11	0.723	0.02	0.876	0.22	0.595
Leotiomycetes, undef. order^	3.2	**5.57**	**0.008**↓	**6.12**	**0.019**↑	**12.55**	**0.001c**
Pseudogymnoascus	1.4	0.21	0.613	**5.02**	**0.022**↑	**8.46**	**0.001c**
**Basidiomycota**	11.9	3.17	0.087↓	2.04	0.168	2.08	0.169
Agaricales	1.6	0.00	0.970	0.92	0.230	0.28	0.533
Amanita fulva	0.52	0.00	0.968	0.05	0.723	0.01	0.929
Cantharellales	0.6	0.07	0.701	0.37	0.464	1.68	0.272
Leucosporidiales	0.6	**4.18**	**0.019**↓	0.20	0.591	0.01	0.925
Russulales	7.7	0.91	0.217	1.38	0.129	1.09	0.184
Russula claroflava	4.5	0.33	0.339	0.23	0.443	0.97	0.229
Russula decolorans	1.26	0.03	0.621	4.29	0.065↑	0.00	0.762
Russula paludosa	0.57	0.12	0.601	0.13	0.632	0.12	0.613
Thelephorales	0.6	1.21	0.070↓	0.24	0.461	0.69	0.269
Pseudotomentella tristis	0.6	0.84	0.091↓	0.19	0.498	0.54	0.327
**Mortierellomycota**	1.6	**6.17**	**0.004**↓	0.52	0.344	0.51	0.343
Mortierella	1.6	5.75	**0.003**↓	0.65	0.267	0.39	0.416
**Mucoromycota**	0.6	3.42	0.084↓	1.32	0.194	0.52	0.339
Umbelopsis	0.6	3.39	0.076↓	1.31	0.190	0.51	0.364

a. relative abundance close to birches in WGR higher than in all others treatments; b. Tree vicinity decreases abundance on WGR, but increases abundance on YGR; c. abundance on YGR > 3 m from birches lower than in all others treatments

^Leotiomycetes with undefined order includes the genera *Pseudogymnoascus* (42% of unknown leotiomycetes), *Meliniomyces* (21%), *Gymnostellatospora* (11%), *Oidiodendron* (10%), *Hyaloscypha* (5%) and *Leptodontidium* (3%)

Table S3 Univariate test results on the impact of summer grazing, tree vicinity, and their interaction on the relative abundance of fungal functional guilds. Arrows indicate increase (↑) or decrease (↓) towards the year-round grazing regime, direction of the significant grazing and tree interaction is noted in footnotes.

	Grazing		Tree		Grazing × Tree	
	$Dev_{1,69}$	*p*	$Dev_{1,68}$	*p*	$Dev_{1,67}$	*p*
**Functional guild**						
Root-associated ascomycetes	1.14	0.205	0.98	0.221	1.04	0.217
Root-associated basidiomycetes	1.88	0.138	2.03	0.115	1.28	0.214
Moulds	**10.19**	**0.002**↓	0.94	0.236	0.41	0.415
Yeasts	**4.37**	**0.020**↓	0.15	0.656	0.01	0.951
Other saprotrophic and litter ass.	0.24	0.541	1.47	0.110	0.04	0.789
Lichenized fungi	**13.40**	**0.002**↑	0.06	0.794	**5.69**	**0.013a**

a. the abundance of lichenized fungi is higher away from trees in WGR, but higher in vicinity of trees in YGR.

Table S4 Univariate test results on the impact of grazing regime, tree vicinity, and their interaction on the relative abundance of most abundant fungal orders and species within the functional guilds. Arrows indicate increase (↑) or decrease (↓) towards the year-round grazing regime or closer vicinity to mountain birch, the directions of significant grazing and tree interactions are noted in footnotes.

Taxa	Rel . abu.	Grazing		Tree		Grazing × Tree	
		$Dev_{1,69}$	*p*	$Dev_{1,68}$	*p*	$Dev_{1,67}$	*p*
**Root-associated ascomycetes**							
Archaeorhizomycetales	16.3	1.61	0.069↑	0.53	0.308	0.01	0.887
Chaetothyriales	10.6	0.09	0.738	0.75	0.358	0.15	0.657
Herpotrichiellaceae	7.9	0.42	0.419	1.71	0.109	0.15	0.633
Chaetothyriales	2.8	0.26	0.402	0.34	0.334	0.05	0.722
Eurotiales (*Elaphomyces.muricatus, EcM)*	0.5	0.55	0.547	0.05	0.686	3.11	0.187
Helotiales	31.4	0.57	0.423	0.06	0.801	1.83	0.129
Pezoloma.ericae (ErM)	13.4	**4.60**	**0.013**↑	0.84	0.267	2.20	0.075
Helotiales, undefined species	10.4	1.46	0.204	0.26	0.591	**3.77**	**0.041a**
Meliniomyces	5.5	0.55	0.381	0.66	0.376	0.99	0.255
Catenulifera	1.7	1.69	0.087↑	0.18	0.600	**6.38**	**0.004b**
Leotiomycetes, undef. order	1.3	**11.11**	**0.001**↓	1.67	0.130	6.37	**0.002c**
**Root-associated basidiomycetes**							
Agaricales	0.8	0.00	0.941	0.20	0.484	0.04	0.770
Amanita.fulva (EcM)	0.5	0.00	0.973	0.05	0.705	0.01	0.916
Russulales	7.7	0.91	0.213	1.38	0.121	1.09	0.166
Russula.claroflava (EcM)	4.5	0.33	0.308	0.23	0.430	0.97	0.190
Russula.decolorans (EcM)	1.3	0.03	0.615	4.29	0.070↑	0.00	0.766
Russula.paludosa (EcM)	0.6	0.12	0.599	0.13	0.642	0.12	0.616
Thelephorales	0.6	1.12	0.071↓	0.22	0.474	0.65	0.285
Pseudotomentella.tristis (EcM)	0.6	0.83	0.093	0.19	0.485	0.55	0.311
**Saprotrophic fungi**							
Agaricales (Other saprotroph)	0.5	0.44	0.285	1.32	0.077	0.17	0.513
Eurotiales (*Penicillium,* mould)	0.6	**4.22**	**0.009**↓	0.48	0.344	0.18	0.561
Leucosporidiales (yeast)	0.6	**4.18**	**0.015**↓	0.20	0.604	0.01	0.929
Mortierellales (mould)	1.6	**6.11**	**0.004**↓	0.55	0.294	0.49	0.365
Mucorales (*Umbelopsis,* mould*)*	0.6	3.43	0.076↓	1.35	0.186	0.52	0.372
**Lichenized fungi**							
Acarosporales	0.7	0.95	0.377	0.01	0.922	1.67	0.244
Acarospora	0.6	0.33	0.623	0.12	0.713	2.22	0.209
Lecanorales	2.6	**13.74**	**0.001**↑	0.00	0.953	2.22	0.072
Cladonia, unidentified.	2.3	**15.82**	**0.002**↑	0.02	0.905	1.90	0.129

a. relative abundance close to birches in WGR higher than in all others treatments; b. Tree vicinity decreases abundance on WGR, but increases abundance on YGR; c. abundance on YGR > 3 m from birches lower than in all others treatments

Table S5 Explanatory value of vectors in the NMDS ordination (*envfit* results).

	r^2	*p*
**Background variables**		
Grazing	0.113	**0.001_{2}**
Tree	0.019	0.282
Block	0.063	0.057
Soil C:N ratio	0.302	**$0.001_{1,2}$**
Soil water content (SWC)	0.205	**0.001_{1}**
SOC kg m^{-2}	0.129	**0.016_{1}**
**Fungal abundance and diversity**		
Fungal abundance (ITS2 copy nr m^{-2})	0.176	**0.002_{1}**
Fungal species richness	0.198	**$0.001_{1,2}$**
Fungal diversity, exp(Shannon–Wiener index)	0.103	**0.031_{1}**
**Fungal guilds**		
Root-associated ascomycetes	0.576	**0.001_{1}**
Root-associated basidiomycetes	0.358	**0.001_{1}**
Moulds	0.645	**$0.001_{1,2}$**
Yeasts	0.228	**0.001_{2}**
Other saprotrophs and litter assoc.	0.108	**0.023_{1}**
Lichenized	0.459	**$0.001_{1,2}$**
**Mycorrhizal and exploration types**		
ErM	0.192	**0.002_{1}**
EcM	0.494	**0.001_{1}**
EcM with simple mycelia	0.502	**0.001_{1}**
EcM, cord formers	0.016	0.591
**Orders within root-associated ascomycetes**		
Archaeorhizomycetales	0.432	**0.001_{1}**
Chaetothyriales (ErM)	0.071	0.082
Eurotiales (*Elaphomyces muricatus*, EcM)	0.091	0.074
Helotiales (ErM)	0.139	**0.004_{1}**
Leotiomycetes, undef. order	0.167	**$0.004_{1,2}$**
Mytilinidiales (ErM)	0.017	0.525
**Orders within root-associated basidiomycetes**		
Agaricales	0.011	0.674
Atheliales	0.011	0.655
Cantharellales (EcM)	0.176	**0.004_{1}**
Russulales (EcM)	0.281	**0.002_{1}**
Sebacinales	0.137	**0.016_{2}**
Thelephorales (EcM)	0.092	**0.045_{1}**
**Saprotophic orders**		
Agaricales	0.005	0.823
Chaetothyriales (mould)	0.079	0.063
Eurotiales (*Penicillium*, mould)	0.380	**$0.001_{1,2}$**
Helotiales	0.123	**0.021_{2}**
Hypocreales	0.013	0.614
Leucosporidiales (yeast)	0.203	**0.002_{2}**
Mortierellales (mould)	0.627	**$0.001_{1,2}$**
Mucorales (mould)	0.283	**0.001a**
Saccharomycetales (yeast)	0.143	**0.004_{2}**
Pezizomycota, undef. order	0.118	**0.016_{1}**
Trechisporales	0.075	0.067
Venturiales	0.045	0.211

Numbers 1 and 2 after significant p-values, indicate whether the variable correlates with NMDS1 or NMDS2 axes or both.

Table S6. Correlations (r^2 values and F-statistics) between the captured ITS2 reads of vegetation and fungal guilds, mycorrhizal types and EcM exploration types. Vegetation is classified into *Betula* species, *Vaccinium* species (i.e. *V. myrtillus* and *V. uliginosum*), other ericaceous species (i.e. *Empetrum hermaphroditum, Phyllodoce caerulea* and *Calluna vulgaris*) and bryophytes (i.e. genera *Polytrichum* and *Dicranum*). Moulds, yeasts and other saprotrophic fungi were pooled into a common group of saprotrophic fungi. Arrows indicate the direction of the correlation (positive ↑ or negative ↓).

	r^2	$F_{1,69}$	*p*
Betula sp.			
Root-associated ascomycetes	0.02	1.24	0.269
Root-associated basidiomycetes	**0.04**	2.80	0.099↑
Saprotrophic fungi	0.00	0.06	0.815
Lichenized fungi	0.00	0.10	0.753
ErM	0.01	1.02	0.317
EcM	**0.09**	6.67	0.012↑
EcM with simple mycelia	**0.06**	4.52	0.037↑
EcM with cord-forming mycelia	**0.08**	5.83	0.018↑
***Vaccinium* sp.**			
Root-associated ascomycetes	**0.12**	9.32	0.003↑
Root-associated basidiomycetes	**0.06**	4.77	0.032↓
Saprotrophic fungi	**0.11**	8.37	0.005↓
Lichenized fungi	0.02	1.58	0.213
ErM	0.03	1.83	0.180
EcM	**0.09**	6.57	0.013↓
EcM with simple mycelia	**0.09**	6.44	0.013↓
EcM with cord-forming mycelia	0.00	0.00	0.952
**Other ericaceaous species**			
Root-associated ascomycetes	**0.25**	23.50	<0.001↑
Root-associated basidiomycetes	**0.14**	11.14	0.001↓
Saprotrophic fungi	**0.20**	16.90	<0.001↓
Lichenized fungi	0.01	0.75	0.390
ErM	**0.09**	7.01	0.010↑
EcM	**0.18**	15.30	<0.001↓
EcM with simple mycelia	**0.17**	14.61	<0.001↓
EcM with cord-forming mycelia	0.02	1.14	0.289
**Bryophytes**			
Root-associated ascomycetes	0.00	0.01	0.927
Root-associated basidiomycetes	0.00	0.00	0.963
Saprotrophic fungi	**0.17**	14.48	<0.001↑
Lichenized fungi	**0.13**	10.65	0.002↓
ErM	0.01	0.70	0.406
EcM	0.00	0.06	0.804
EcM with simple mycelia	0.00	0.00	1.000
EcM with cord-forming mycelia	0.01	0.62	0.434

Table S7 Results of Akaikes' information criteria test on how well fungal abundance, functional guilds and taxonomy (i.e. fungal orders complemented with significantly correlating genera and species only) explain soil organic carbon stocks. The table shows the following metrics: the number of estimated parameters (K), the Akaikes' information criteria adjusted for sample size (AICc), the difference in AICc relative to the best model (ΔAICc), the weight of evidence for this model among the candidate models (AICcWt), the cumulative Akaike weights (CumWt) and the model's maximum likelihood estimation (log-likelihood, LL).

	K	AICc	ΔAICc	AICcWt	CumWt	LL	
**Fungal abundance**							
ITS2 copy nr g^{-1} DW	3	299.35					*↑
ITS2 copy nr m^{-2}	3	300.11					*↑
**Functional guilds**							
Saprotrophic fungi	3	287.30	0	0.99	0.99	-140.47	***↓
Root-assoc. ascomycetes	3	297.94	10.64	0	1	-145.79	**↑
Lichenized	3	302.29	14.99	0	1	-147.97	
Root-assoc. basidiomycetes	3	302.50	15.20	0	1	-148.07	
**Fungal orders**							
Thelephorales	3	291.57	0	0.42	0.42	-142.61	***↓
Mucorales	3	292.07	0.5	0.33	0.75	-142.86	***↓
Sebacinales	3	294.86	3.29	0.08	0.84	-144.25	**↓
Sordariomycota, undef. order	3	295.65	4.08	0.06	0.89	-144.65	**↓
Archaeorhizomycetales	3	296.69	5.12	0.03	0.92	-145.16	**↑
Ascomycota, undef. order	3	297.37	5.8	0.02	0.95	-145.51	**↓
Saccharomycetales	3	298.1	6.53	0.02	0.96	-145.87	*↓
Mortierellales	3	298.45	6.88	0.01	0.98	-146.05	*↓
Leucosporidiales	3	301.04	9.47	0	0.98	-147.34	′↓
Eurotiales	3	301.07	9.5	0	0.98	-147.36	′↓
Venturiales	3	302.15	10.58	0	0.99	-147.9	
Chaetothyriales	3	302.55	10.98	0	0.99	-148.1	
Lecanoromycetes, undef. order	3	302.98	11.41	0	0.99	-148.31	
Acarosporales	3	303.11	11.54	0	0.99	-148.38	
Lecanorales	3	303.16	11.59	0	0.99	-148.4	
Russulales	3	303.57	12	0	0.99	-148.61	
Agaricales	3	303.87	12.3	0	0.99	-148.75	
Mytilinidiales	3	303.88	12.31	0	1	-148.76	
Cantharellales	3	304.26	12.69	0	1	-148.95	
Helotiales	3	304.3	12.73	0	1	-148.97	
Trechisporales	3	304.32	12.75	0	1	-148.98	
Hypocreales	3	304.39	12.82	0	1	-149.01	
Pezizomycota, undef. order	3	304.44	12.87	0	1	-149.04	
Atheliales	3	304.46	12.89	0	1	-149.05	
Leotiomycetes, undef. order	3	304.54	12.97	0	1	-149.09	
**Fungal genera / species^**							
Penicillium (Eurotiales)	3	290.78	0	0.52	0.52	-142.21	***↓
Umbelopsis (Mucorales)	3	292.16	1.38	0.26	0.78	-142.9	***↓
Pseudotomentella tristis (Thelephorales)	3	293.89	3.11	0.11	0.88	-143.76	**↓
Serendipita (Sebacinales)	3	295.16	4.38	0.06	0.94	-144.4	**↓

Archaeorhizomyces (Arhaeorhizomycetales)	3	297.04	6.26	0.02	0.96	-145.34	**↑
Russula claroflava (Russulales)	3	297.95	7.17	0.01	0.98	-145.8	*↓
Mortierella (Mortierellales)	3	298.09	7.31	0.01	0.99	-145.87	*↓
Russula decolorans (Russulales)	3	299.13	8.35	0.01	1	-146.39	*↑

^For fungal genera and species, only significantly ($p < 0.05$) correlating groups are shown.
Asterisks indicate significance of F-statistics, where *** $p < 0.001$, ** $p < 0.01$, * $p < 0.05$, and ′ $p < 0.1$; the direction of the correlation (positive ↑ or negative ↓) is indicated with arrows.

Table S8 Summary of best explanatory value models on how fungal abundance, functional guilds, and taxonomy explain SOC when the individual and/or interacting effect of grazing regime (G) and/or tree vicinity (T) is included. This table summarizes the variables included to the best model, as well as the Akaikes' information criteria adjusted for sample size (AICc), adjusted r^2 value and F-statistics of the best model. Only those fungal groups that improved model fit, in compared to own effects of grazing and tree, are shown.

	Best model	AICc	adj. r^2	F_{df}	*p*
**Fungal abundance**					
ITS2 copy nr g^{-1} DW	T:copynr	299.18	0.08	$3.85_{2,68}$	0.026n.s.
ITS2 copy nr m^{-2}	T:copynr	299.88	0.07	$3.48_{2,68}$	0.036a
**Functional guild**					
Root-assoc. ascomycetes	guild + T	**295.23**	**0.13**	$6.02_{2,68}$	0.004↑
Root-assoc. basidiomycetes	guild + T	**300.39**	**0.06**	$3.22_{2,68}$	0.040↓
Saprotrophic fungi	guild + T	**285.96**	**0.23**	$11.60_{2,68}$	<0.001↓
Lichenized	T:guild	**296.25**	**0.11**	$5.45_{2,68}$	0.006b
**Fungal orders**					
Acarosporales	order + T + G	300.98	0.07	$2.71_{3,67}$	0.052↓
Archaeorhizomycetales	order + T	293.44	0.15	$7.04_{2,68}$	0.002↑
Ascomycota, undef. order	order + G	297.27	0.10	$4.89_{2,68}$	0.010↓
Chaetothyriales	order + T + G	300.88	0.05	$2.79_{2,68}$	0.068↑
Eurotiales	order + T	299.39	0.07	$3.74_{2,68}$	0.029↓
Hypocreales	G:order	298.74	0.08	$4.09_{2,68}$	0.021c
Lecanorales	G + G:order	294.36	0.15	$5.16_{3,67}$	0.003d
Leucosporidiales	order + T	299.71	0.07	$3.57_{2,68}$	0.034↓
Mortierellales	order + T	296.65	0.11	$5.23_{2,68}$	0.008↓
Mucorales	order + T	289.41	0.19	$9.43_{2,68}$	<0.001↓
Saccharomycetales	order + T	297.20	0.10	$4.92_{2,68}$	0.010↓
Sebacinales	G + G:order	290.08	0.20	$6.87_{3,67}$	<0.001e
Sordariomycota, undef. order	order + T	294.49	0.13	$6.44_{2,68}$	0.003↓
Thelephorales	order + T	291.24	0.17	$8.33_{2,68}$	0.001↓
Trechisporales	G + G:order	299.88	0.08	$3.10_{3,67}$	0.032e
Venturiales	G + G:order	300.92	0.07	$2.73_{3,67}$	0.050e
**Fungal genera / species^**					
Archaeorhizomyces	genera +T	293.82	0.14	$6.82_{2,68}$	0.002↑
Mortierella	genera + T	296.12	0.11	$5.52_{2,68}$	0.006↓
Penicillium	genera + T	288.46	0.20	$10.02_{2,68}$	<0.001↓
Pseudotomentella tristis	genera + T	293.57	0.15	$6.96_{2,68}$	0.002↓
Russula claroflava	genera + T + G	295.45	0.14	$4.74_{3,67}$	0.005↓
Russula decolorans	genera + G	298.04	0.09	$4.46_{2,68}$	0.015↑
Serendipita	G + G:genera	291.39	0.19	$6.34_{3,67}$	0.001e
Umbelopsis	genera + T	289.54	0.19	$9.36_{2,68}$	<0.001↓

^For fungal genera and species, only significantly ($p < 0.05$) correlating groups are shown.
In case of own effect of copy nr, order or function, the direction of correlation is indicated by an arrow
In case the best model included an interaction between with grazing or tree, a post-hoc test was conducted for grazing regimes / birch vicinities separately. Post hoc results are indicated by the following numbers: n.s. correlation not significant when birch vicinities tested separately; a. positive correlation on birch plots; b. negative correlation on plots < 3 m from trees; c. positive correlation under YGR; d. negative correlation on YGR and close to significant (P=0.051) positive correlation under WGR; e. negative correlation under YGR
