# Supplementary material for: Reindeer control over subarctic treeline alters soil fungal communities with potential consequences for soil carbon storage
Source: Glob Chang Biol. 2021 Jun 14;27(18):4254–68. doi: 10.1111/gcb.15722 (PMC13420783; doi:10.1111/gcb.15722)
Supplement: Supplementary file 2 — Supplementary Material [file GCB-27--s002.pdf]

Supplementary Method description for "Reindeer control over subarctic treeline alters soil fungal communities with potential consequences for soil carbon storage" by Henni Ylänne, Rieke L. Madsen, Carles Castaño, Daniel B. Metcalfe and Karina E. Clemmensen

In this Supplementary Method description, we present the most common plant species documented in the study area, and compare this cover and biomass data to the ITS2-derived data on plant abundance with regression analyses.

Data collection and the comparison approach

Differences among grazing regimes in plant cover and biomass were estimated from the original coordinates, moving max 1 m aside in order to not be directly under mountain birch. Cover of *Betula pubescens* was estimated from a radius of 3.99 m (50 m^2), whereas the cover of vascular species, and bryophytes and lichen genera was estimated from a 0.5 × 0.5 m square (0.25 m^2). Only species with a cover higher than 0.1% were noted. To assess ground-layer biomass, a representative area of 0.19 × 0.19 m^2 within the cover square was selected, and all living biomass was collected therein and dried in 100 °C before weighing. As in the assessment of fungal communities, we were particularly interested to map out the role of mountain birch in driving fungal community changes, the sampling design for collecting soil samples for this study did not align with the vegetation assessment. From the original coordinates (Fig. S2 in the separate Supplementary Figure and Table file), we moved on average 3 m (max 20 m) to the nearest area with at least 3 m distance to any mountain birch. Similarly, we moved on average 33 m (max 150 m) to the closest non-sampled mountain birch to each plot. As the soil samples derived under the birch were further away from the cover and biomass plots, here we only compare the plant read data from non-birch soil samples to the cover and biomass data.

Here, we firstly report how the dominant species in the area were captured by the ITS2 primers (Table M1). Secondly, we compare the ITS2-derived metric of plant abundance to the plant cover on the original coordinates (Table M2). We do the comparison by two means: 1) by treating each soil sample and cover plot as an individual replicate, and 2) by pooling the data within blocks and grazing regimes (n = 6). In the first approach, two samples with more than 10 m distance between the cover plot and soil sampling location were omitted from the analyses, resulting in a sample size of 33. Correlation between ITS2 based plant abundance data and ground-layer biomass is only reported within blocks and grazing regimes.

Comparison of ITS2 derived plant abundance data with the recorded plant cover and biomass

The ITS2 primers captured the majority of the plant species recorded on the study area, accounting for 94.4 ± 2.9% of the plant cover recorded in the blocks (mean ± SD; Table M1). Of the species with a cover higher than 1%, only *Vaccinium vitis-idaea* (average cover 1.85 ± 1.31) was not captured (Table M1).

The ITS2-derived estimate on plant abundance correlated positively with the recorded cover of *B. czerepanovii*, *Vaccinium myrtillys, V. uliginosum, Empetrum nigrum* ssp. *hermaphroditum, Phyllodoce caerulea, Calluna vulgaris* and *Dicranum* spp. when comparing the cover of the closest coordinates to the DNA abundance in the closest soil sample (Table M2). Instead, no correlations between *Polytrichum* sp. and *Cladonia* DNA abundance and cover were noted (Table M2). When looking at block averages, high within block variation in the abundance of most common species likely hindered the detection of patterns between blocks (Table M2). Yet, correlations were found among the more rare species, i.e. *V. uliginosum*, *P. caerulea, C. vulgaris* and *Polytrichum* spp. (Table M2).

Dicranum and *Polytrichum* DNA abundance correlated significantly with the biomass of these within the blocks ($F_{1,4} = 37.56$, $p = 0.004$, $r^2 = 0.90$ for *Dicranum* sp. and $F_{1,5} = 14.93$, $p = 0.012$, $r^2 = 0.75$ for *Polytrichum* sp.), whereas no positive correlation was observed between *Cladonia* biomass and *Cladonia* DNA abundance.

Table M1. Average plant cover in the in the study area (averaged over the two grazing regimes and the three blocks therein; listed in the order of abundance) and the capturing of each species / genera by the ITS2 primers.

**Plant species**	**Cover (%)**	**Captured by ITS2-primers**
Empetrum nigrum ssp. *hermaphroditum*	26.1	yes
Cladonia sp.	22.9	yes
Betula nana	14.0	yes*
Calluna vulgaris	8.4	yes
Vaccinium myrtillus	5.7	yes
Dicranum sp.	4.4	yes
Phyllodoce caerulea	3.5	yes
Stereocaulon sp.	2.9	yes
Polytrichum sp.	2.2	yes
Betula pubescens ssp. *czerepanovii*	2.0	yes*
Avenella flexuosa	1.9	yes
Vaccinium vitis idaea	1.9	no
Vaccinium uliginosum	0.9	yes
Arctostaphylos alpina	0.9	yes
Juncus trifidus	0.7	no
Cetraria sp.	0.7	yes
Agrostis stolonifera	0.4	no
Carex bigelowii	0.4	no
Lycopodium sp.	0.1	no
Hylocomnium splendens	0.1	yes
Nephroma arcticum	0.1	yes
Andromeda polifolia	<0.1	yes
Diphasiastrum alpinum	<0.1	no
Kalmia procumbens	<0.1	yes
Solidago virgaurea	<0.1	no
Trientalis europeae	<0.1	no
Rubus chamaemorus	<0.1	yes

*Captured ITS2 reads of *Betula* were not identified to species, and may thus include only *B. pubescens*, *B. nana* or both.

Table M2. F-statistics and r^2-values of the correlations between ITS2-derived plant abundance data and the visual estimates of plant cover from the original coordinates. On the left, the ITS2-derived plant abundance in samples > 3 m away is compared to the cover on the original coordinates (n = 33). On the right, the abundance and cover data have been pooled within blocks / grazing regime (n = 6).

	Closest coordinates (n = 33)			Within blocks / grazing reg. (n = 6)		
	$F_{1,31}$	*p*	r^2	$F_{1,4}$	*p*	r^2
Betula pub. ssp. *czerepanovii*	**5.49**	**0.026**	0.15	1.15	0.344	0.22
Emp. nig. ssp. *hermaphroditum*	**13.24**	**0.001**	0.30	0.03	0.867	0.01
Phyllodoce caerulea	**9.64**	**0.004**	0.24	**10.46**	**0.032**	0.72
Calluna vulgaris	**31.39**	**<0.001**	0.50	**10.36**	**0.032**	0.72
Vaccinium myrtillus	**5.90**	**0.021**	0.16	2.82	0.168	0.41
Vaccinium uliginosum	**27.02**	**<0.001**	0.47	**15.20**	**0.018**	0.79
Dicranum spp.	**7.91**	**0.008**	0.20	1.31	0.317	0.25
Polytrichum spp.	1.45	0.237	0.04	**7.71**	**0.050**	0.66
Cladonia spp.	0.53	0.472	0.02	0.48	0.525	0.11
